# *WDR45* variants cause ferrous iron loss due to impaired ferritinophagy associated with nuclear receptor coactivator 4 and WD repeat domain phosphoinositide interacting protein 4 reduction

**DOI:** 10.1093/braincomms/fcac304

**Published:** 2022-11-23

**Authors:** Kiwako Tsukida, Shin-ichi Muramatsu, Hitoshi Osaka, Takanori Yamagata, Kazuhiro Muramatsu

**Affiliations:** Department of Pediatrics, Jichi Medical University, Tochigi 329-0498, Japan; Division of Neurological Gene Therapy, Jichi Medical University, Tochigi 329-0498, Japan; Center for Gene & Cell Therapy, The Institute of Medical Science, The University of Tokyo, Tokyo 108-8639, Japan; Department of Pediatrics, Jichi Medical University, Tochigi 329-0498, Japan; Department of Pediatrics, Jichi Medical University, Tochigi 329-0498, Japan; Department of Pediatrics, Jichi Medical University, Tochigi 329-0498, Japan

**Keywords:** SENDA/BPAN, *WDR45*/WIPI4, iron metabolism, NCOA4, ferittinophagy

## Abstract

Static encephalopathy of childhood with neurodegeneration in adulthood/β-propeller protein-associated neurodegeneration is a neurodegenerative disorder with brain iron accumulation caused by the variants of *WDR45*, a core autophagy-related gene that encodes WD repeat domain phosphoinositide interacting protein 4. However, the pathophysiology of the disease, particularly the function of *WDR45*/WD repeat domain phosphoinositide interacting protein 4 in iron metabolism, is largely unknown. As no other variants of core autophagy-related genes show abnormalities in iron metabolism, the relation between autophagy and iron metabolism remains to be elucidated. Since iron deposition in the brain is the hallmark of static encephalopathy of childhood with neurodegeneration in adulthood/β-propeller protein-associated neurodegeneration, iron chelation therapy has been attempted, but it was found to worsen the symptoms; thus, the establishment of a curative treatment is essential. Here, we evaluated autophagy and iron metabolism in patient-derived cells. The expression of ferritin and ferric iron increased and that of ferrous iron decreased in the patient cells with *WDR45* variants. In addition, the expression of nuclear receptor coactivator 4 was markedly reduced in patient-derived cells. Furthermore, divalent metal transporter 1, which takes in ferrous iron, was upregulated, while ferroportin, which exports ferrous iron, was downregulated in patient-derived cells. The transfer of *WDR45* via an adeno-associated virus vector restored WD repeat domain phosphoinositide interacting protein 4 and nuclear receptor coactivator 4 expression, reduced ferritin levels, and improved other phenotypes observed in patient-derived cells. As nuclear receptor coactivator 4 mediates the ferritin-specific autophagy, i.e. ferritinophagy, its deficiency impaired ferritinophagy, leading to the accumulation of ferric iron-containing ferritin and insufficiency of ferrous iron. Because ferrous iron is required for various essential biochemical reactions, the changes in divalent metal transporter 1 and ferroportin levels may indicate a compensatory response for maintaining the intracellular levels of ferrous iron. Our study revealed that the pathophysiology of static encephalopathy of childhood with neurodegeneration in adulthood/β-propeller protein-associated neurodegeneration involves ferrous iron insufficiency via impaired ferritinophagy through nuclear receptor coactivator 4 expression reduction. Our findings could aid in developing a treatment strategy involving *WDR45* manipulation, which may have clinical applications.

## Teaser

Tsukida *et al*. found WD repeat domain 45 variants cause deficiency of nuclear receptor coactivator 4, which mediates ferritinophagy in the autophagy-related neurodegenerative disease, static encephalopathy of childhood with neurodegeneration in adulthood/β-propeller protein-associated neurodegeneration. Impaired ferritinophagy results in ferric iron accumulation within ferritin and damages cellular function by ferrous iron deficiency. Gene transfer rescues these phenotypes.

## Graphical Abstract

**Graphical abstract:**
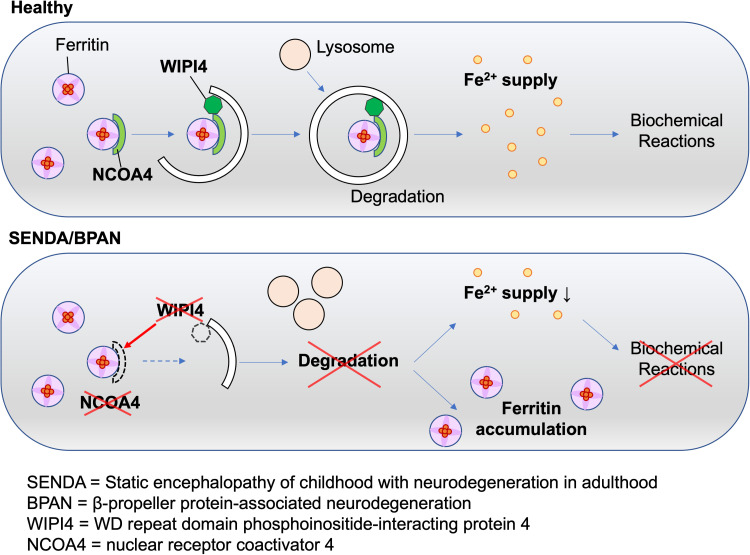

## Introduction

Static encephalopathy of childhood with neurodegeneration in adulthood (SENDA)/β-propeller protein-associated neurodegeneration (BPAN) (OMIM #300894) is a neurodegenerative disease caused by a de novo mutation of WD repeat domain 45 (*WDR45*) located on Xp 11.23. Clinical findings from patients with SENDA/BPAN have demonstrated that psychomotor retardation occurs in early childhood, and motor and cognitive dysfunctions, such as dystonia and extrapyramidal signs, develop when patients are in their 20s and early 30s. Subsequently, the symptoms rapidly progress, and the patients are rendered bedridden. SENDA/BPAN is a neurodegeneration with brain iron accumulation (NBIA) subtype, which is characterized by iron deposits in the substantia nigra and globus pallidus. These deposits are observed based on brain MRI and brain pathological findings.^1–4^ Since the identification of the causative gene in 2012, there have been advancements in diagnosis and SENDA/BPAN accounts for 40–45% of all NBIA subtypes at present.^5^ However, the pathophysiology of SENDA/BPAN remains largely unknown, which poses a significant challenge for developing effective treatment strategies.

The causative gene of SENDA/BPAN is *WDR45* coding WIPI4, which is known to play an important role in autophagy.^2,4^ Autophagy is an essential intracellular degradation system that is involved in quality control and amino acid supply in cells, as unnecessary intracellular proteins or organelles are degraded and removed by lysosome. Briefly, when a cell is starved of amino acids, a lipid membrane called the isolation membrane is formed near the endoplasmic reticulum (ER). This isolation membrane engulfs proteins or organelles and forms autophagosome. The autophagosome fuse with lysosome to develop into autolysosome, and their contents are degraded by lysosomal enzymes.^6–8^ WIPI4 is involved in the formation and elongation of isolation membrane^9–13^; however, there is no direct evidence of its involvement in iron metabolism. To the best of our knowledge, abnormalities in other major autophagy-related genes do not lead to impaired iron metabolism. Hence, it is unclear why variants of *WDR45* induce impairments in iron metabolism.

Iron is an essential molecule for various cellular biochemical reactions, and it exists in two forms in the cell, namely, ferrous iron (Fe^2+^) and ferric iron (Fe^3+^). Among these, ferrous iron is highly reactive and is involved in various cellular biochemical reactions, i.e. oxidative phosphorylation, metabolite synthesis, and oxygen transport. However, excess ferrous iron can produce reactive oxygen species (ROS) via the Fenton reaction, resulting in cytotoxicity. Therefore, iron homeostasis is tightly regulated.^14–17^ Transferrin receptor (TfR) and divalent metal transporter 1 (DMT1) are responsible for intracellular iron uptake. The only molecule responsible for iron export from the cell is ferroportin (FPN).^16^ Excess iron is stored in ferritin as ferric iron in non-redox and stable state.^18,19^ When the demand for ferrous iron increases in the cell owing to biochemical reactions, ferritin is degraded by autophagy and the ferrous iron is released to cytoplasm. The ferritin-specific autophagy is called ferritinophagy, wherein a cargo receptor called nuclear receptor coactivator 4 (NCOA4) binds ferritin heavy chain and directs ferritin to the isolation membrane.^20,21^ The iron metabolism in SENDA/BPAN evaluated in previous studies was insufficient and inconclusive, as the analyses did not distinguish between ferrous iron and ferric iron and investigated only some of the molecules related to iron uptake, efflux, and storage, or used culture cell lines that overexpress a mutant of *WDR45* under non-physiological conditions.^22–24^ In addition, no studies have focused on ferritinophagy. It is still elusive which point of iron metabolism is affected by *WDR45* variants.

Here, we evaluated autophagic activity, iron metabolism, and the molecules involved in iron homeostasis, including ferritinophagy, using fibroblasts derived from healthy participant and patients with SENDA/BPAN. We aimed to elucidate the distinct mechanisms involved in the dysregulation of intracellular iron homeostasis of SENDA/BPAN arising because of *WDR45* variants. Additionally, we aimed to restore the obtained phenotypes via *WDR45* gene transfer. Our findings, which revealed the dysregulation of iron metabolism caused by *WDR45* variants, provide novel insights into the relationship between intracellular iron deposition and cytotoxicity and could aid in the development of fundamental treatments.

## Materials and methods

### Participants

We analyzed four Japanese patients with SENDA/BPAN whose diagnosis was confirmed using genetic screening. All clinical summaries and genetic analyses are shown in Table 1. Two *WDR45* transcripts were registered in the RefSeq database, and all four variants harboured the common exons of the two transcripts. Human WIPI4 protein comprises 361 aa (NP_009006.2 from NM_007075.4) and seven WD repeat domains according to UniProt (Q9Y484).

**Table 1:** Clinical features of patients with SENDA/BPAN along with *WDR45* variants

Patient (reference)		1	2	3^4^	4^25^
Age, gender		14y, Boy	41y, Woman (deceased)	35y, Woman	10y, Girl
Variant		c.130 + 2T > C (p.Gly28_Leu43del)	c.19C > T (p.Arg7*)	c.516G > C (p.Asp174Valfs*29)	c.830 + 1G > A
CADD PHRED		33	36	24.7	33
XCI	Hapll/HhaI	100:0/100:0	98:2/97:3	84:16/97:3	99:1/99:1
**Neurological symptoms**					
Current status		Bedridden, Spastic quadriparesis	Bedridden	Wheelchair	Walking with support
Initial symptoms		Global developmental delay	Psychomotor retardation	Psychomotor retardation	General developmental delay
Initial walking		Not acquired	1y6mo	2y7mo	3y
Speech ability		Non-verbal	Three words	One word	Non-verbal
Cognitive dysfunction during childhood		Static	Static	Static	Static
Start of cognitive decline		-	25y	25y	-
Period until bedridden after decline		From birth	5y	-	-
Dystonia		-	+	+	-
Parkinsonism		Bilateral positive Babinski sign	Rigidity	Rigidity, Akinesia	-
Progressive dementia during adulthood		NA	+	+	NA
Psychiatric symptoms		-	None	Aggressive behaviours	Hyperthymia
Epileptic seizure		From 2y FIAS	FBTCS	+	From 2y gelastic seizure
Autonomic symptom		-	Bradycardia	Hypothermia, cold extremity	Hypothermia, cold extremity
Stereotyped behaviour		-	-	+	+
**Radiological features**					
MRI	Iron deposition	GP, SN	GP, SN	GP, SN	GP, SN
	Cerebral atrophy	Remarkable at 39y	Moderate at 25 and 27y	Remarkable at 39y	Mild at 6y,
	Cerebellar atrophy	Mild at 39y	Mild at 25 and 27y	Mild at 39y	NA
CT findings		Reduced white matter volume enlarged the lateral ventricles	Reduced white matter volume enlarged the lateral ventricles	Mild high density in SN	NE
**Neurophysiological examinations**					
EEG		Bilateral anterior focal slow waves with spike	Low voltage	Bilateral frontal spike/low voltage/slow wave	Bilateral front-temporal polyspikes and waves
EMG		NE	Dystonic pattern	NE	NE

CADD, Combined Annotation-Dependent Depletion (http://cadd.gs.washington.edu/score): PHRED scores of 10–20 and >20 are regarded as deleterious and the 1% most deleterious, respectively; XCI, X-chromosome inactivation analysis; EEG, electroencephalogram; EMG, electromyogram; FIAS, focal impaired awareness seizure; FBTCS, focal to bilateral tonic-clonic seizure; GP, globus pallidus; MB, midbrain; MRI, magnetic resonance imaging; SN, substantia nigra; NA, not applicable; NE, not examined; y, years; mo, months.

### Mutation screening

Mutation screening of exons 3–12 covering the *WDR45* coding region (of transcript variant 1, GenBank accession NM_007075.4) was performed by direct sequencing. PCR was performed with a 20 μl of the mixture containing 1 μl of genomic DNA, 2 μl of 10× PCR buffer, 2 μl of dNTPs, 0.5 μM of each primer and 1 U of Taq polymerase (Takara, R001A). Details of the PCR conditions and primer sequences are given in Supplementary Table 1.^4^

### X-inactivation analysis

The X-inactivation pattern was studied using the human androgen receptor (HUMARA) assay as previously described.^4^ Briefly, genomic DNA was extracted from the participants, and the control fibroblasts were digested with two methylation-sensitive enzymes, *Hpa*II and *Hha*I. Fluorescently-labeled products were analyzed using the FASMAC Co., Ltd. X-inactivation ratios of less than or equal to 80:20 were considered to represent a random pattern, ratios higher than 80:20 were considered to represent a skewed pattern, and ratios higher than 90:10 were considered to represent a markedly skewed pattern. Details of the PCR conditions and primer sequences are provided in Supplementary Table 2.^4^

### Cell culture and media

Skin samples were obtained via skin biopsies from four patients with SENDA/BPAN. Written informed consent was obtained from their caregivers. Control fibroblasts were purchased from PromoCell Company (#C-12300, Heidelberg, Germany) and used as a male control (normal human dermal fibroblast). Wild-type (WT) and *ATG5* knock-out (KO) HeLa cells were kindly provided by N. Mizushima from the University of Tokyo. We used fibroblasts derived from passages 4–12 for assays. Cells were maintained in growth medium [Dulbecco's modified Eagle's medium (DMEM) (Gibco, 11885) with 10% foetal bovine serum (Gibco) and 1% penicillin/streptomycin (Gibco, 15140-163)] at 37°C in a humidified atmosphere containing 5% CO_2_/95% air. For the starvation stimuli, cells were washed twice with PBS and incubated in amino acid-free DMEM (Wako, 048-33575). For lysosome inhibition, 100 nM of bafilomycin A1 (Baf A1) (BioViotica, BVT-0252M001) was used. All experiments were performed using male fibroblasts as the control.

### Retroviral infections and generation of stable cell lines

The plasmids used in this study (pMRX-IP-GFP-LC3-RFP, pMRX-IP-WDR45-EGFP, and pMRX-IP-FLAG-NCOA4) were kindly gifted by N. Mizushima from the University of Tokyo. Each plasmid was transformed into *E. coli* DH5α (TaKaRa, 9057) and amplified. The plasmids were extracted using the QIAGEN Plasmid Kit (QIAGEN, 12125 or 12143) according to the manufacturer’s instructions, and the sequences were confirmed using the Sanger method. HEK293T cells (purchased from RIKEN BRC) were transiently transfected with each plasmid along with pCG-VSV-G and pCG-gag/pol (kindly gifted by T. Yasui, National Institutes of Biomedical Innovation, Health and Nutrition, Osaka, Japan) using the Lipofectamine 3000 reagent (Thermo Fisher, L300015). Three days after infection, the virus-containing supernatant was collected and concentrated by centrifugation (2330 × *g*, 20 min) using a dialysis filter (Amicon Ultra-15; Merck, UFC 910008). Fibroblasts were incubated with the collected virus-containing medium and 4 μg/ml of polybrene (Nacalai Tesque, 12996-81) for 8 h and 2 μg/ml of polybrene for 1 day. Uninfected cells were removed using 1 ng/μl of puromycin (InvivoGen: ant-pr-1).^26^ Single clones were isolated using the limiting dilution technique.

### Determination of cellular autophagy flux in fibroblasts expressing GFP-LC3-RFP

Autophagic activity was determined using a previously described method.^26,27^ Fibroblasts transfected with this probe were seeded in 96-well plates, at a density of 3 × 10^4^ cells/well, and cultured to 90% confluence. After amino acid starvation with or without Baf A1 treatment, the cells were fixed with 4% paraformaldehyde (PFA) in PBS for 15 min at 25°C. Then, the cells were washed twice with PBS, and intracellular fluorescence intensities of the GFP and RFP were measured using a multimode plate reader (EnVision, Perkin Elmer) with excitation/emission at 460/535 nm and 550/620 nm, respectively. Intracellular fluorescence intensities of the GFP and RFP were measured using a laser fluorescence microscope (BZ-X810, Keyence) with excitation/emission at 470/525 nm and 545/605 nm, respectively. The GFP/RFP ratio was used to estimate the autophagic activity by summing the absolute value of changes in GFP/RFP during the starvation conditions with or without Baf A1.

### Production of adeno-associated virus vectors

The adeno-associated virus (AAV) vector plasmid comprised an expression cassette consisting of the cytomegalovirus (CMV) immediate early promoter, the cDNA of human *WDR45* (Gene ID: 11152) and the simian virus 40 polyadenylation signal sequence between the inverted terminal repeats (ITRs) of the AAV3 genome. AAV9 vp cDNA was synthesized, and the sequence was identical to that previously described,^28^ except for the substitution of thymidine for adenine 1337. This introduced a change in the amino acid at position 446 from tyrosine to phenylalanine.^29^ Recombinant AAV vectors were produced by transient transfection of HEK293 cells with the vector plasmid, AAV3 rep, tyrosine-mutant AAV9 vp expression plasmid, and the adenoviral helper plasmid pHelper (Agilent Technologies, Santa Clara, CA), as described previously.^30^ The recombinant viruses were purified following their isolation from two sequential continuous CsCl gradient centrifugation steps, and the viral titres were determined by qPCR. The vectors were prepared at a titre of 2.3× 10^11^ vg/μl.

### *In vitro* transduction

Fibroblasts were infected with 5 × 10^5^ vg/cell of AAV-WDR45. Three days after infection, the cells were harvested and used for the downstream assay.

### Immunoblotting

Total cell lysates were obtained using MPER buffer (Thermo Fisher, 78501) in the presence of protease (Roche, 11873580001) and phosphatase (Roche, 04906845001) inhibitors. Cells were incubated in the lysis buffer on ice for 5 min, and the samples were centrifuged at 14,000 × *g* for 10 min at 4°C, and the supernatant was collected. Protein concentrations were measured using a bicinchoninic acid protein assay kit (Takara, T9300A) according to the manufacturer’s instructions. Then, an equal amount of protein was separated using sodium dodecyl sulphate-polyacrylamide gel electrophoresis and transferred to polyvinylidene difluoride membranes (Thermo Fisher, IB24001) using iBlot 2 (Thermo Fisher). Membranes were incubated in blocking buffer (5% dried skimmed milk dissolved in PBS containing 0.1% Tween-20; PBS-T) followed by overnight incubation at 4°C with appropriate antibodies diluted in antibody dilution buffer (1% dried skimmed milk in PBS-T). The following day, the membranes were incubated for 1 h at 25°C with secondary horseradish peroxidase antibodies diluted in antibody dilution buffer. Reactive bands were detected using a western blotting detection reagent (Takara, T7103A or Thermo Fisher, A38554). Images were captured and quantified using an Amersham Imagermager (GE Healthcare). The band intensity was normalized to that of the endogenous controls, and the protein levels were compared between the healthy and patient cells by normalizing with the average protein levels in the healthy cells. The primary and secondary antibodies used are listed in Supplementary Table 3.

### Quantitative real-time PCR

Total RNA was extracted from fibroblasts using TRIzol Reagent (Thermo Fisher, 15596018) according to the manufacturer’s instructions, as previously described.^31^ Total RNA was quantified using a NanoDrop 8000 (Thermo Fisher). Then, 300–700 ng of RNA was reverse-transcribed to cDNA using SuperScript IV VILO Master Mix (Thermo Fisher, 11756500). Quantitative real-time PCR was performed using TaqMan Gene Expression Master Mix (Thermo Fisher, 4369016) and 1 μl of cDNA. PCR programmes were run on a QuantStudio® 3 Real-Time PCR System (Applied Biosystems, Life Technologies, Carlsbad, CA) with the following steps: initial incubation at 50°C for 2 min followed by 95°C for 10 min, followed by 40 cycles of 15 s at 95°C and 1 min at 60°C. The mRNA expression levels were calculated using the 2-ΔΔCt method and normalized to the geometric mean of the ΔCt of the housekeeping gene, human glyceraldehyde-3-phosphate dehydrogenase (*hGAPDH*). The TaqMan probes are listed in Supplementary Table 4.

### Immunofluorescence

Fibroblasts were cultured on glass coverslips (Matsunami, C012001) to 70–80% confluence. Cells were fixed with 4% PFA/PBS for 15 min at 25°C and then washed with PBS. Cells were permeabilized with 0.2% Triton-X100 in PBS or 50 μg/ml digitonin (Wako, 043-21376) in PBS for 5 min at 25°C. Cells were then washed with PBS and blocked with blocking buffer (5%NGS in PBS) at 25°C for 1 h. After blocking, the cells were incubated with a primary antibody in blocking buffer at 4°C overnight. Cells were then washed with PBS and later incubated with the appropriate Alexa Fluor-conjugated secondary antibody at 25°C for 1 h. After washing with PBS, coverslips were mounted on the glass slides with ProLong Glass (Thermo Fisher, P36981), and fluorescent images were acquired using a laser fluorescence microscope (BZ-X810, Keyence). The primary and secondary antibodies are listed in Supplementary Table 5.

### Evaluation of the intracellular ferrous iron content

To determine the intracellular ferrous iron content, FerroOrange (Dojindo, F374) was used according to the manufacturer's protocol. Fibroblasts were seeded in 96-well plates at 3 × 10^4^ cells/well, cultured to 70–80% confluence, and washed twice with PBS. The cells were then treated with 1 μM FerroOrange and 1 μM Hoechst 33342 (DOJINDO, 346-07951) in HBSS (Wako, 085-09355) for 30 min at 37°C. The cells were fixed with 4% PFA/PBS for 15 min at 25°C. After washing with PBS, fluorescence intensities were captured using a multimode plate reader (EnVision, Perkin Elmer) and fluorescence images were captured using a fluorescence microscope (BZ-X810, Keyence).

### Modified Berlin blue staining

Ferric iron accumulation was examined using modified Berlin blue staining. Cells were cultured on glass coverslips until 70–80% confluence. The cells were fixed in 4% PFA/PBS for 15 min at 25°C. The cells were then permeabilized with 50 μg/ml of digitonin in PBS for 5 min at 25°C. After 6 h incubation with 2% potassium ferrocyanide mixed with 2% HCl (Wako, 296-21541), ferrous iron content was visualized after 2 h of incubation with DAB (Nacalai Tesque, 25985-50) and metal enhancer for the DAB stain (Nacalai Tesque, 07388-24). The dehydrated cells were mounted onto slides using balsam.

### Extra cellular flux analysis

Cells were seeded at a density of 3 × 10^4^ cells/well in XFp cell culture plates (103022-100, Agilent Technologies) and cultured for 24 h. Cells were treated with four drugs—oligomycin, carbonyl cyanide p-trifluoromethoxy-phenylhydrazone, antimycin A, and rotenone. Oxygen consumption rate (OCR) measurements were performed using an extracellular flux analyzer (Seahorse XFp, Agilent Technologies).

### Statistical analysis

Statistical analyses were performed using the JMP pro16. Statistical differences between control groups and experimental groups were determined using *P*-values via non-parametric comparisons with Wilcoxon test or Tukey’s HSD test using data obtained from three or more independent experiments. Data are shown as the mean ± standard error of the mean (SEM).

### Ethical agreement

This research was approved by the Bioethics Committee for Human Gene Analysis and Gene Recombination Research at Jichi Medical University (approval number 21–93 and 20–12), and written informed consent was obtained from all patients’ caregivers because all patients had no ability of cognition (Informed consent of control fibroblasts was waived since they were purchased from cell bank).

## Results

### *WDR45*/WIPI4 expression was lacking in male and deficient or decreased in female patients

**Figure 1:**
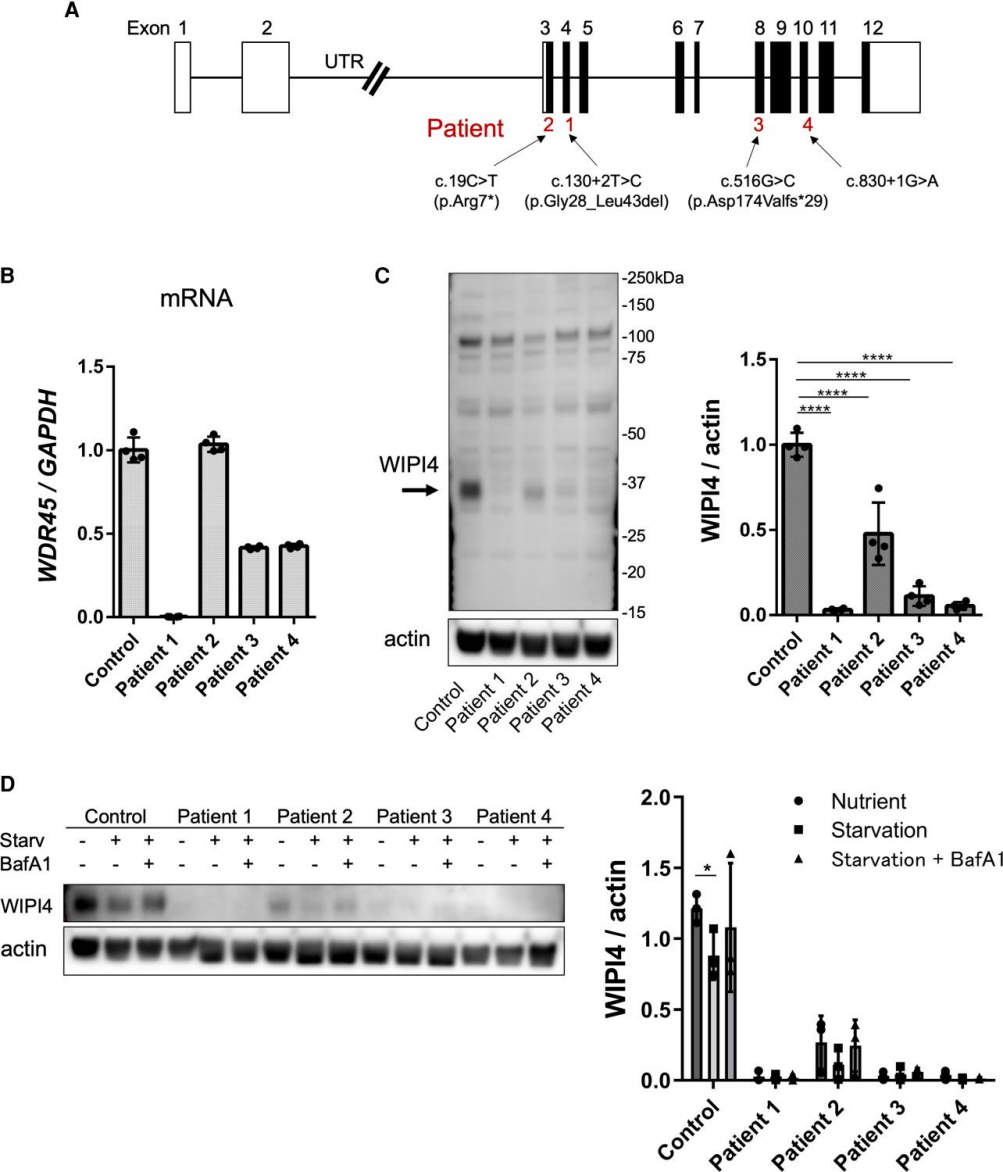
**Fibroblast *WDR45*/WIPI4 expression under conditions involving the availability of nutrients and lysosomal inhibitory conditions.** (**A**) An illustration of *WDR45*, which comprises 12 exons (rectangles). The UTRs and coding regions are shown in white and black, respectively. Three variants were confirmed as *de novo* (patients 1, 3 and 4); the other could not be confirmed as the parental samples were unavailable (patient 2). (**B**) *WDR45* mRNA expression ratio evaluated via qRT-PCR using TaqMan. The expression ratio was normalized to that of the endogenous *hGAPDH*. (**C**) WIPI4 protein expression was confirmed using immunoblotting. Actin was used as the loading control. A comparison of the quantitative expression ratio to that of the control is shown. A one-way ANOVA with Tukey’s HSD test was used for analysis (*n* = 4, control versus Patients 1–4; ****P < 0.0001) (Supplementary material for uncropped blots). (**D**) The protein expression of WIPI4 was analyzed using immunoblotting under nutrient condition and starvation conditions with or without Baf A1 treatment. Actin was used as the loading control. The quantitative expression ratio compared to that of the control is shown. One-way ANOVA with Wilcoxon test was used for analysis (*n* = 3, *P = 0.0049). All data are represented as the mean ± SEM of a minimum of three independent experiments (Supplementary material for uncropped blots).

The clinical features of four patients (three females and one male) with SENDA/BPAN harbouring *WDR45* variants are summarized in Table 1. Genetic variants of *WDR45* are shown in Fig. 1A. The transcript-level expression of *WDR45* and the protein expression of WIPI4 were evaluated using skin fibroblasts derived from four patients and the healthy participant. *WDR45* mRNA was not detected in male Patient 1; however, the expression observed in female Patient 2 was similar to that observed in the healthy control. The expression observed in female Patients 3 and 4 was ∼50% of that observed in the healthy control (Fig. 1B). First, we confirmed the ability of the available antibodies to detect WIPI4 before the study (Supplementary Figs. 1 and 2). We used antibodies from Proteintech for the experiments. Patients 1, 3 and 4 showed reduced expression of WIPI4, while Patient 2 exhibited ∼50% of the WIPI4 expression observed in the healthy control (Fig. 1C). The expression of *WDR45* mRNA was examined under conditions of starvation with or without Baf A1, a molecule that inhibits lysosomal degradation over time. Under both conditions, the mRNA levels of *WDR45* increased over time and were absent in Patient 1 during both conditions (Supplementary Fig. 3). The protein levels of WIPI4 were analyzed using immunoblotting. The expression decreased under starvation conditions but was recovered with Baf A1 treatment (Fig. 1D). To confirm the intracellular localization of WIPI4, immunofluorescence was performed using organelle and autophagy markers. WIPI4 partially colocalized with the KDEL motif, which is an ER marker. WIPI4 also colocalized with the lysosomal marker lysotracker under nutrient and starvation conditions. Additionally, WIPI4 colocalized with LC3 and partially colocalized with p62 under conditions of starvation with Baf A1 treatment, indicating that WIPI4 was localized on autophagosome. WIPI4 did not colocalize with mitochondria (Supplementary Fig. 4).

### Autophagy was inhibited in patient cells

**Figure 2:**
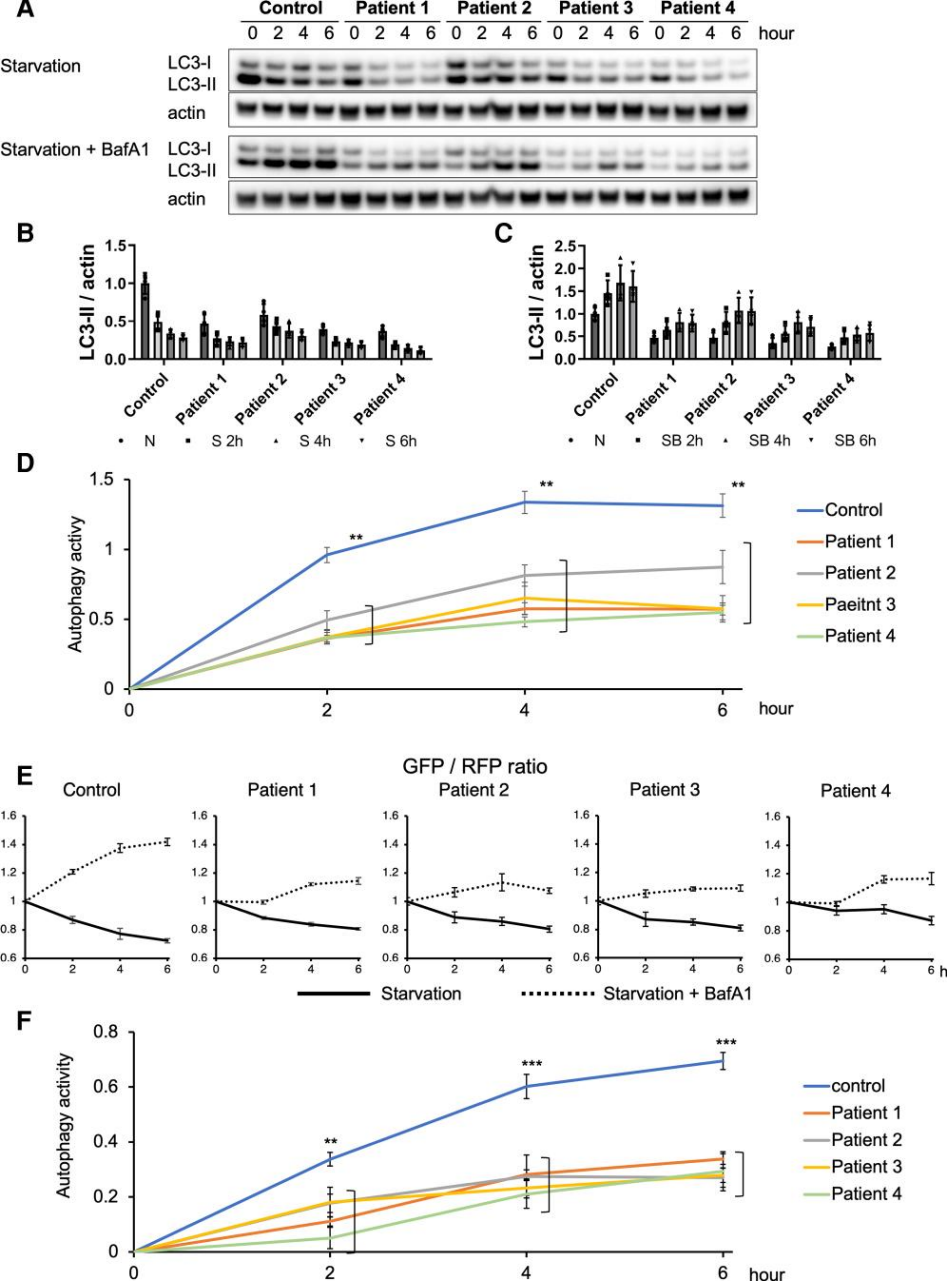
**Autophagic activity in patient fibroblasts was reduced to ∼50% of that in the healthy control. (A)**The levels of LC3-I and II under starvation conditions with or without Baf A1 treatment were evaluated for the indicated intervals using fibroblasts. LC3-II was rapidly degraded in control fibroblasts; however, its degradation was suppressed in patient fibroblasts under starvation conditions. Moreover, LC3-II accumulation was increased in control fibroblasts; however, it was suppressed in patient fibroblasts under starvation conditions with Baf A1 treatment. Actin was used as the loading control. Data are representative of a minimum of three independent experiments (Supplementary material for uncropped blots). **(B, C)** Quantitative expression ratio under starvation conditions with or without Baf A1 treatment. N, nutrient condition; S, starvation condition; SB, starvation condition with Baf A1 **(D)** Autophagic activities were calculated as follows: LC3-II of starvation + Baf A1 (Fig. 2c) minus LC3-II of starvation (Fig. 2b) for the same time. Autophagic activities in patient fibroblasts were significantly decreased compared to those in the control fibroblasts at all indicated times. Data are represented as mean ± SEM of three or more experiments. One-way ANOVA with Wilcoxon test was used for analysis. (*n* = 3, 2 h; **P = 0.0029, 4 h; **P = 0.0029, 6 h; **P = 0.0029) **(E)** Fibroblast stable single clones expressing the GFP-LC3-RFP probe were incubated in starvation conditions with or without Baf A1 treatment over time. The fluorescence of GFP and RFP was measured using a multimode plate reader. GFP/RFP ratios were plotted over time under starvation conditions with or without BafA1 treatment. **(F)** The sum of the GFP-LC3 degradation and accumulation ratios in each fibroblast were plotted for the indicated times. Autophagy activities were significantly decreased compared with those observed in control fibroblasts at all indicated times. One-way ANOVA with Wilcoxon test was used for statistical analysis. (*n* = 6, 2 h; **P = 0.0010, 4 h; ***P = 0.0005, and 6 h; ***P = 0.0001) Data are represented as the mean ± SEM of a minimum of three independent experiments.

We evaluated autophagic activity in fibroblasts by quantifying the changes in the LC3-II expression under starvation conditions with or without Baf A1 treatment (Fig. 2A–C). The degradation of LC3-II in patients reduced to ∼40–60% of that in the healthy participant (Fig. 2D). p62, a major autophagy substrate, showed a similar pattern of changes (Supplementary Fig. 5). Furthermore, we used the fluorescent probe, GFP-LC3-RFP, to evaluate autophagic activity as previously mentioned.^26,27^ This probe is cleaved by endogenous ATG4 proteases into equimolar amounts of GFP-LC3 and RFP. GFP-LC3 is degraded by autophagy, while RFP remains in the cytosol (serving as an internal control). Hence, the GFP/RFP signal ratio is inversely correlated with autophagic activity. The GFP/RFP ratio reduced over time under conditions of starvation and was enhanced under conditions of starvation with Baf A1 treatment in control cells. In contrast, the GFP/RFP ratio remained relatively static during both conditions in patient cells (Supplementary Fig. 6). Quantification of autophagic activity in patient cells over time—using the fluorescent probe GFP-LC3-RFP—is shown under conditions involving starvation with and without Baf A1 treatment. The difference in the GFP/RFP ratio was greater in control cells at each time point (Fig. 2E). In summary, the autophagic activity in the patient cells decreased to ∼50% of that observed in the healthy control over time (Fig. 2F). The data evaluated using two different analyses demonstrate the same results for autophagic activity.

### Ferric iron and ferritin content increased, and ferrous iron content decreased in patient cells

Although ferrous iron plays an important role in biochemical reactions, previous studies have mainly evaluated the total iron content in the cell.^23,24^ Herein, we measured the levels of intracellular ferrous iron using the fluorescent reagent FerroOrange and those of ferric iron using Berlin blue staining.

**Figure 3:**
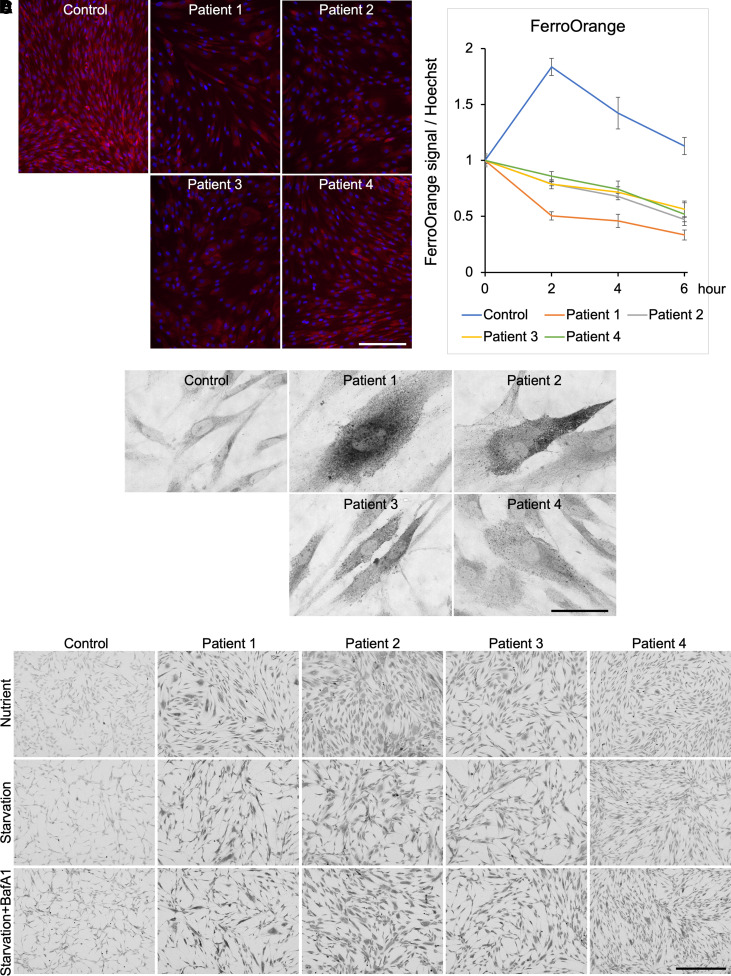
**Microscopic imaging of the intracellular ferrous and ferric iron.** (**A**) Fibroblasts were incubated with 1 μM FerroOrange for 30 min. RFP fluorescence was attributed to the intracellular ferrous iron content. The nuclei were stained blue using Hoechst staining. Ferrous iron contents were lower in patient fibroblasts than in the control fibroblasts. Scale bar, 200 μm. Statistical analyses are shown in Supplementary Fig. 7. **(B)** FerroOrange signals normalized by Hoechst 33342 measured using a multimode plate reader under starvation conditions were plotted for all fibroblasts over time. Ferrous iron content increased rapidly and then decreased in the control fibroblast. However, the levels of ferrous iron only decreased in patient fibroblasts (*n* = 4). (**C**) Fibroblasts were subjected to Berlin blue staining for the detection of ferric iron. Ferric iron was more densely stained in patient fibroblasts than in the control fibroblasts. Scale bar, 50 μm. (**D**) Fibroblasts were stained via Berlin blue staining under conditions involving the availability of nutrients and starvation conditions with or without Baf A1 treatment for 8 h. Notably, the enhanced staining in Baf A1-treated fibroblasts relative to that under other conditions was observed only in the control fibroblasts. Berlin blue staining intensity decreased upon starvation and increased under starvation conditions with Baf A1 treatment in the control fibroblasts. However, the signals corresponding to starvation conditions with or without Baf A1 treatment showed little change in the patient fibroblasts. Scale bar, 500 μm. Statistical analyses are shown in Supplementary Fig. 7.

The fluorescence of FerroOrange was weaker in the patient cells than that in the cells of the healthy participant, indicating that patients had low amounts of ferrous iron (Fig. 3A, Supplementary Fig. 7A). The FerroOrange fluorescence observed under starvation conditions was plotted against time. The fluorescence signal increased during early starvation and then decreased during late starvation in the control fibroblasts. In contrast, the fluorescence decreased unidirectionally under starvation conditions over time in patient fibroblasts (Fig. 3B). In Berlin blue staining, the patient cells were more intensely stained, indicating an increase in the ferric iron content (Fig. 3C). Moreover, ferric iron levels decreased under conditions of starvation in control cells because of ferritin degradation and increased under starvation conditions with Baf A1 treatment because of disturbed ferritin degradation in the control cells. However, the levels of ferric iron remained unchanged under both conditions in all patient cells (Fig. 3D, Supplementary Fig. 7B).

Mitochondria use iron to maintain their functions, for example, electron transport, oxidative phosphorylation, and heme synthesis. To determine the effect of ferrous iron insufficiency on mitochondrial function, OCRs were measured using an extracellular flux analyzer. Basal respiration, adenosine triphosphate (ATP) production, maximum respiration and spare capacity were reduced in patient cells (Supplementary Fig. 8).

**Figure 4:**
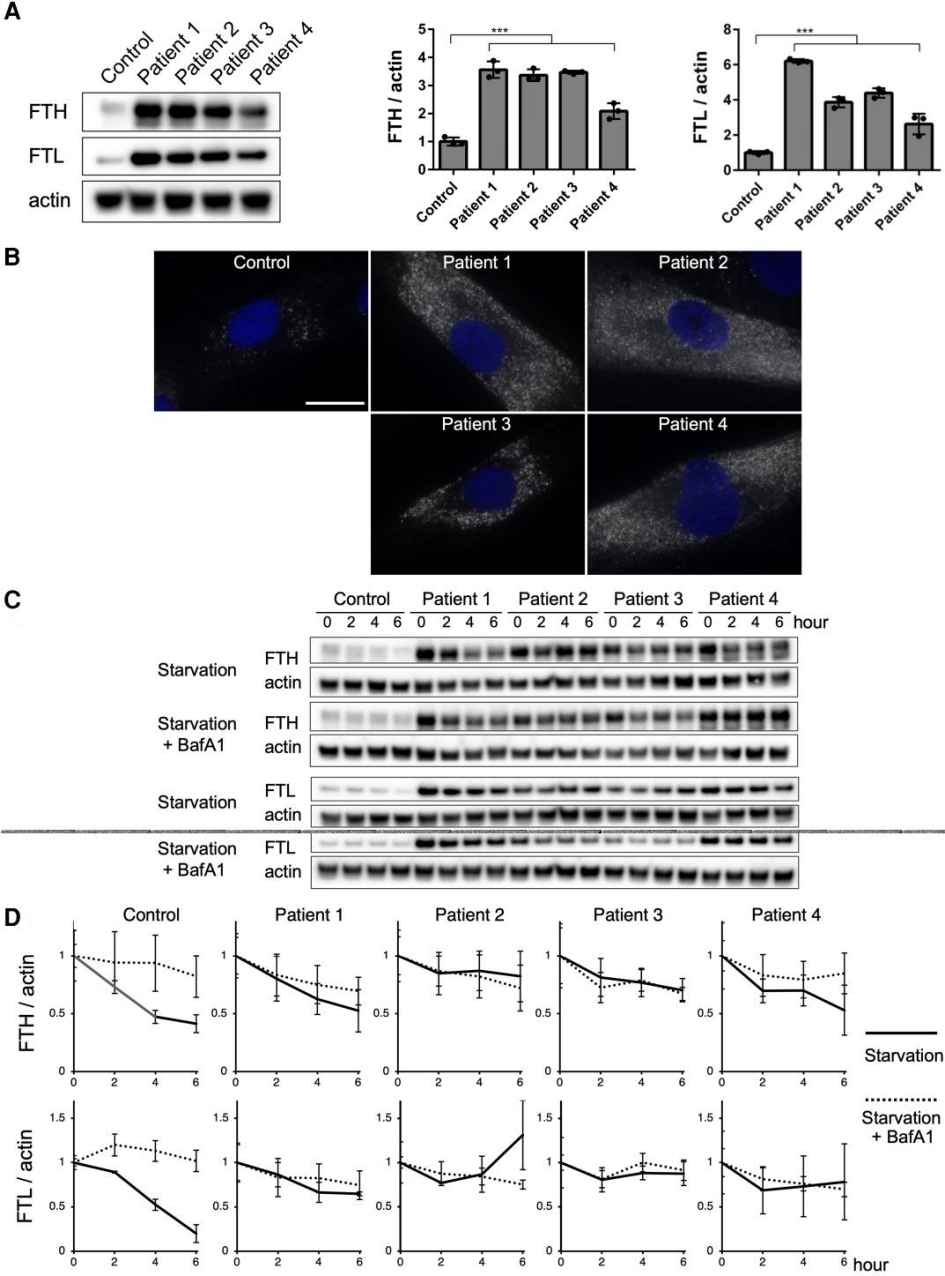
**Immunoblotting and immunofluorescence analyses of ferritin heavy and light chains.** (**A**) Ferritin heavy and light chains were significantly accumulated in patient fibroblasts, as examined with immunoblotting using whole cell lysates prepared from fibroblasts. Actin was used as the loading control. One-way ANOVA with Wilcoxon test was used for analysis. (*n* = 3, FTH; **P = 0.0095, FTL; **P = 0.0095) (Supplementary material for uncropped blots). (**B**) Immunofluorescence indicated that ferritin accumulated robustly in patient fibroblasts. Nuclei were stained with DAPI. Scale bar, 20 μm. (**C**) Immunoblotting of ferritin heavy and light chains under starvation conditions with or without Baf A1 treatment demonstrated that the ferritin heavy and light chains were degraded under starvation over time, and the degradation was inhibited under starvation conditions with Baf A1 treatment in the control fibroblasts. However, the degradation of ferritin heavy and light chains was minimal in all patient fibroblasts under both conditions. Actin was used as the loading control (Supplementary material for uncropped blots). (**D**) The degradation rates of the ferritin heavy and light chains detected by immunoblotting were plotted over time. The degradation rates rapidly decreased under starvation conditions and were stable under starvation conditions with Baf A1 treatment in the control fibroblasts. However, the degradation rates were nearly stable under both conditions in patient fibroblasts (*n* = 3). Data are represented as mean ± SEM of values from three experiments.

In cells, ferric iron is stored within ferritin, therefore, we analyzed the expression of the ferritin protein. Ferritin consists of a heavy chain and a light chain. The heavy chain oxidizes ferrous iron to ferric iron, which is stored in the light chain.^18,19^ Immunoblotting showed that the levels of the heavy and light chains markedly increased in patient cells (Fig. 4A). In addition, immunofluorescence revealed strong fluorescence corresponding to ferritin expression in the patient cells (Fig. 4B). In contrast, the mRNA expressions of ferritin heavy and light chains were not significantly different between the control and patients (Supplementary Fig. 9). Next, the changes in ferritin protein expression over time were examined under starvation conditions with or without Baf A1 treatment. In the cells of the healthy participant, ferritin levels decreased rapidly upon starvation and remained almost unchanged in starvation combined with Baf A1 treatment. In contrast, the ferritin levels in all patient cells remained static or mildly decreased under both conditions (Fig. 4C). These changes were plotted based on the protein expression levels observed at the beginning of the treatment as a reference. In the cells of the healthy participant, the difference between the two conditions was significant. However, in patient cells, the graphs of the two conditions were similar to each other, with minor differences (Fig. 4D). It is noteworthy that ferritin could not be degraded in the patient cells, even though their autophagy activity was ∼50% of that of the cells of healthy participants.

### The expression of the NCOA4 protein required for ferritinophagy was downregulated in patient cells

**Figure 5:**
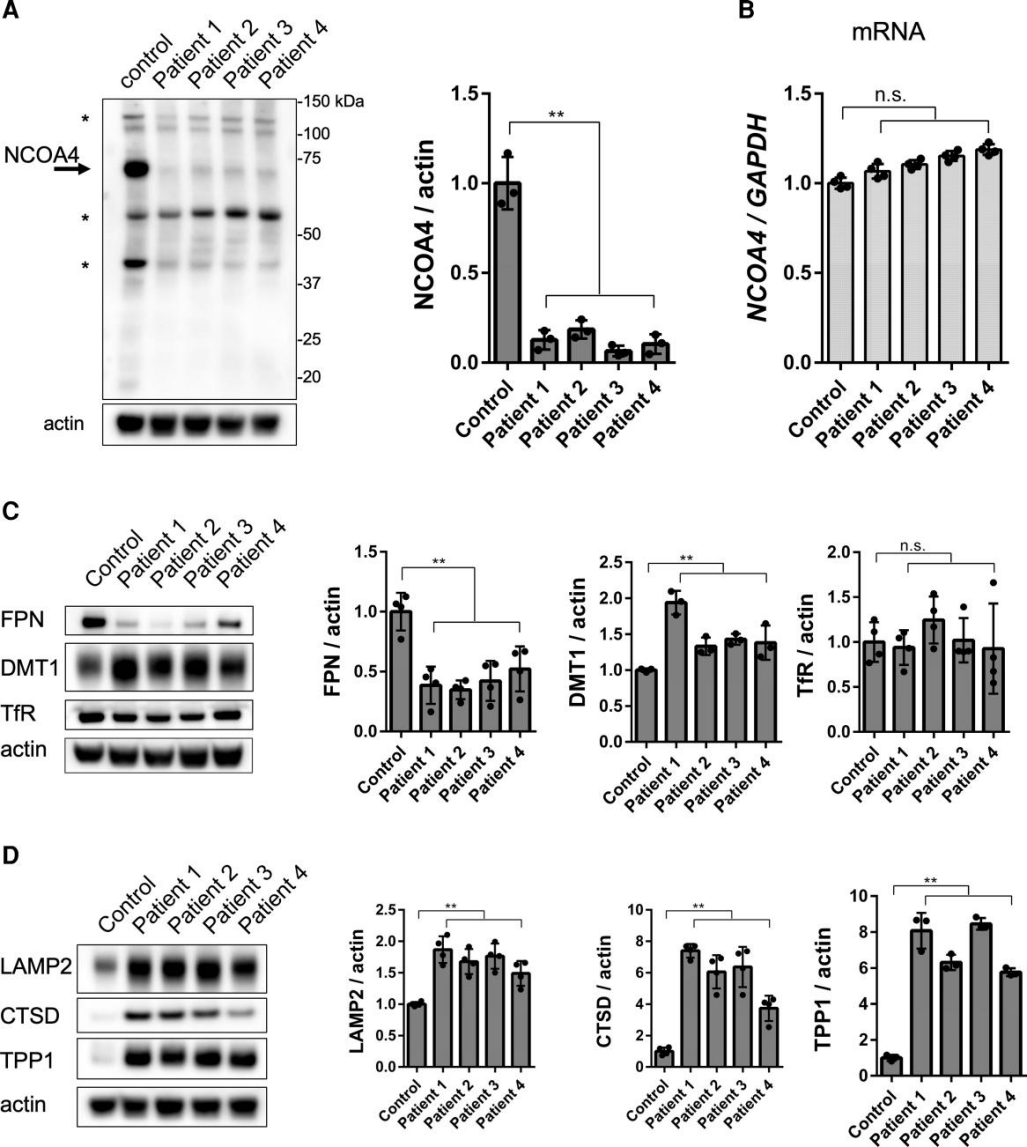
**NCOA4 protein expression was markedly reduced in all patient fibroblasts. Protein levels of molecules involved in iron influx/outflux, and lysosomes were altered in patient fibroblasts.** (**A**) Immunoblot analysis demonstrated that the NCOA4 expression was markedly reduced under nutrient-availability conditions in all patient fibroblasts. Asterisks indicate non-specific immunoreactive bands. Actin was used as the loading control (Supplementary material for uncropped blots). (**B**) The *NCOA4* mRNA was detected following qRT-PCR using the TaqMan probe. The expression ratio was normalized to that of endogenous *hGAPDH*. (**C**) FPN protein expression decreased and DMT1 protein expression increased in patient fibroblasts. No differences were observed in the expression of TfR protein between control and patient fibroblasts (Supplementary material for uncropped blots). (**D**) The levels of lysosomal proteins, membrane protein LAMP2, and major lysosomal enzymes CTSD and TPP1 were increased notably in all patient fibroblasts (Supplementary material for uncropped blots). Actin was used as the loading control. Quantitative expression ratios compared to those observed in the control patient are shown. Data are represented as mean ± SEM of a minimum of three experiments. A one-way ANOVA with Wilcoxon test was used for analysis. *P*-values are shown in Supplementary Table 6. n.s., not significant.

Despite the residual autophagic activity, ferritin degradation was significantly decreased under starvation conditions leading to ferritin accumulation in all patient cells. Therefore, we confirmed alteration in molecules related to ferritin degradation. The specific degradation of ferritin by autophagy is known as ferritinophagy.^20,21,32^ Surprisingly, the expression of NCOA4, a cargo receptor of ferritin, was markedly reduced in the patient cells in normal nutrient conditions (Fig. 5A). In contrast, the expression levels of *NCOA4* mRNA were maintained (Fig. 5B). To investigate the localization of NCOA4, immunofluorescence was performed using control fibroblasts transfected with WDR45-EGFP via a retrovirus. NCOA4 was distributed throughout the cytoplasm and colocalized with WIPI4 under normal nutrient conditions (Supplementary Fig. 10).

### FPN expression decreased and that of DMT1 increased in the patient cells

To investigate the changes in the intracellular iron content in the patient cells, we examined the molecules involved in iron influx and efflux. FPN expression was significantly reduced in the patient cells compared to that observed in the control cells from healthy participant, indicating that the release of ferrous iron was inhibited. Furthermore, DMT1 levels were elevated in the patient cells, indicating that ferrous iron uptake was promoted. No changes were observed in the expression of TfR (Fig. 5C).

### Expression of lysosomal membrane proteins and enzymes increased in patient cells

WIPI4 is involved in the formation of isolation membranes,^9–13^ which is the initial step in autophagy; hence the early stages of autophagy are impaired in SENDA/BPAN. To investigate how this affects lysosome, the destination site of the autophagy pathway, we quantified lysosomal protein expression by immunoblotting. In all patient cells, the expression levels of the lysosomal membrane protein lysosome-associated membrane protein 2 (LAMP2) and the lysosomal enzymes cathepsin D (CTSD) and tripeptidyl peptidase 1 (TPP1) were increased compared with those in healthy control cells because of impaired lysosomal degradation, as reflected by the reduction in autophagic activity (Fig. 5D).

### *WDR45* gene transfer restored its phenotype, including NCOA4 expression

**Figure 6:**
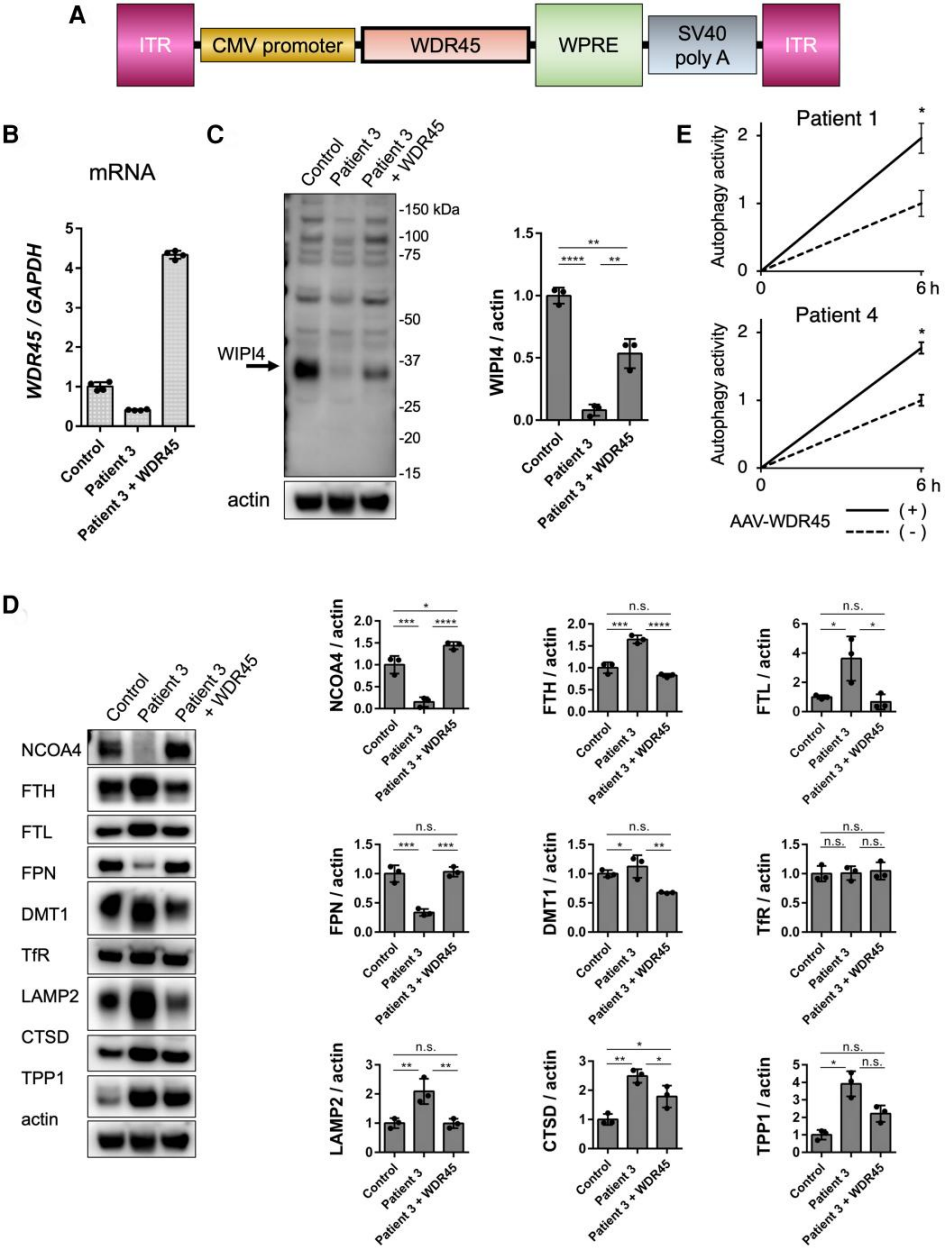
***WDR45* gene transfer by AAV vector restored autophagic activity and related phenotypes of patients.** (**A**) Illustration of the vector constructs. The expression cassette consisted of CMV promoter, human *WDR45* complementary DNA, woodchuck hepatitis virus posttranscriptional regulatory element (WPRE), and simian virus (SV) 40 poly A signal inserted between the ITR of AAV3. The capsids were tyrosine-mutant AAV9. The vectors were prepared at a titre of 2.3× 10^11^ vg/μlL. **(B)** The *WDR45* mRNA expression ratio was determined by qRT-PCR using the TaqMan probe. The expression ratio was normalized to that of endogenous *hGAPDH*. **(C)** WIPI4 protein expression after *WDR45* gene transfer using AAV-WDR45 was confirmed via immunoblotting. Actin was used as the loading control (Supplementary material for uncropped blots). **(D)** NCOA4 protein expression was completely restored by the *WDR45* gene transfer. Moreover, FTH, FTL, FPN, DMT1, LMP2, CTSD, and TTP1 expression levels were restored compared to those observed in control fibroblast. Actin was used as the loading control. The quantitative expression ratio compared to the control is shown. A one-way ANOVA with Tukey’s HSD test was used for analysis. *P*-values are shown in Supplementary Table 7. n.s., not significant (Supplementary material for uncropped blots). **(E)** The autophagic activity was measured using patient 1 and 4 fibroblasts expressing the GFP-LC3-RFP probe. The autophagic activities were increased approximately twice and improved compared to those observed in the control fibroblasts following *WDR45* gene transfer using AAV-WDR45. A one-way ANOVA with Wilcoxon’s test was used for analysis (Patient 1; *P = 0.037, Patient 4; *P = 0.012). All data are represented as mean ± SEM of values obtained from a minimum of three experiments.

To confirm the effect of restoring *WDR45* expression, we performed *WDR45* gene transfer into the cells of Patient 3 using an AAV vector. An illustration of AAV9/3-WDR45, which contains an expression cassette consisting of the CMV immediate early promoter, cDNA of human *WDR45*, and the simian virus 40 polyadenylation signal sequence between the ITRs is shown in Fig. 6A. *WDR45* mRNA expression increased approximately 4-folds compared with that observed in the healthy control, whereas WIPI4 expression was restored to ∼50% of that in the healthy cells (Fig. 6B and C). In addition, NCOA4 expression was completely restored to the level observed in the healthy cells, and the amount of ferritin was significantly decreased. The expression levels of proteins involved in the iron transfer, such as DMT1 and FPN, and lysosomal proteins, such as LAMP2, CTSD, and TPP1, were also improved to the same levels as those observed in the control cell (Fig. 6D). Finally, autophagic activity, which was ∼50% of that observed in the control cells, was restored up to 2-fold after gene transfer using the AAV vector, which indicated that autophagic activity in the cells of patients 1 and 4 transfected with GFP-LC3-RFP probe improved to the level observed in control cells (Fig. 6E). Conversely, gene transfer of FLAG-NCOA4 to cells of Patient 3 with retrovirus restored the protein expression of NCOA4 but did not resolve the ferritin accumulation (Supplementary Fig. 1^1^).

### ATG5-deficient cells did not show changes in iron and ferritin levels

Since ferritinophagy refers to ferritin-specific autophagy, if ferritinophagy and autophagy share a common pathway, it is likely that ferritin will accumulate when the major autophagy gene is lacking. Thus, we examined iron-related molecules in HeLa cells deficient in *ATG5*, a major autophagy gene. In *ATG5*KO cells, there were no significant changes in ferritin heavy and light chain, ferrous iron, ferric iron, and NCOA4 levels (Supplementary Fig. 1^2^).

## Discussion

There are many isolated cases of NBIA,^33^ and the brain iron accumulation may or may not be a primary consequence of a defective gene. To establish the involvement of pathways that explain more clearly the relationship between iron and BPAN is important because this may indicate that brain iron accumulation is a direct consequence of a defect in *WDR45*. In patients with SENDA/BPAN, iron deposition is observed in the basal ganglia,^1^ suggesting that *WDR45* is involved in iron metabolism. However, which part of iron metabolism is disturbed and what mechanisms lead to cellular damage, remain unknown. In addition, although *WDR45* is an autophagy-related gene,^4,34,35^ it is unclear how the autophagic functions of *WDR45* are associated with iron metabolism.

In this study, we clarified three significant points in SENDA/BPAN pathophysiology. First, the impairment of iron metabolism is due to impaired ferritinophagy induced by NCOA4 deficiency based on the decreased expression of WIPI4. Second, the relationship between iron metabolism and autophagy can be attributed to the impaired ferritinophagy in the background of *WDR45* variants. Finally, the cause of cytotoxicity is ferrous iron insufficiency based on disturbed ferritinophagy. The major limitation of this study is the use of a single control. However, *WDR45* gene transfer restored the protein expression of WIPI4 as well as the other phenotypes in one of the patient-derived cells. Therefore, the obtained patient phenotypes are attributed to the lack of *WDR45*.

Iron exists as both ferrous and ferric irons. Ferrous iron is used in many biochemical reactions, such as mitochondrial respiration, DNA synthesis, oxygen transportation, myeline synthesis and neurotransmitter synthesis.^17,36^ However, excess ferrous iron produces ROS via the Fenton reaction, which leads to severe cytotoxicity.^37^ Therefore, iron availability is tightly regulated within cells. TfR and DMT1 are responsible for intracellular iron uptake and FPN is responsible for iron export.^38^ To protect cells from cytotoxicity, excess iron is sequestered in the ferritin form as ferric iron, which is a redox-inactive form.^18,19^ When iron demand increases for cell viability, ferritin is degraded by autophagy, which releases iron into the cytosol as ferrous iron.^39^ In addition, ferritin consists of a heavy chain and a light chain. The heavy chain oxidizes ferrous iron into ferric iron, which is stored in the light chain.^19^ In patient cells, ferritin and ferric iron levels were markedly increased, while those of ferrous iron was decreased. In addition, FPN expression was decreased, while that of DMT1 increased. When ferritinophagy was evaluated based on the differences between autophagy induction by starvation and inhibition by Baf A1 treatment, ferritinophagy was found to be significantly decreased. These results suggest that the ferritin degradation was impaired in patient cells, resulting in the accumulation of ferritin and ferric iron and a reduction in the release of ferrous iron into the cytoplasm. The changes observed in DMT1 and FPN expression are compensatory responses that promote ferrous iron uptake and inhibit its release. This suggests that the expression levels of iron transporters are altered to compensate for the lack of intracellular ferrous iron in the patient cells. Previous studies using patient-derived fibroblasts have detected elevation of total iron and ferrous iron. Therefore, excess iron was considered to be the cause of pathogenesis of SENDA/BPAN.^22,23^ However, our study demonstrated the decrease in ferrous iron and the accumulation of ferric iron due to the inability to degrade ferritin in the patient cells. Xiong *et al.*^24^ argued that autophagy deficiency leads to accumulation of TfR, resulting in iron overload, and the excess amount of ferrous iron produces ROS, which causes cellular damage. Their study used HeLa or HEK293T cells overexpressed with WT or mutant *WDR45*. It differs from our experiments in cell types. Also, the function of endogenous *WDR45* remains in cells with forced expression. Therefore, their study may be evaluating phenomena other than that occurring in the patient cells. Ferrous iron is required for various biochemical reactions, such as myelin synthesis and neurotransmitter synthesis.^17,36^ Therefore, cytotoxicity, especially neuronal loss that is observed in the patient's cerebrum and basal ganglia, would be induced by ferrous iron insufficiency.

We next investigated the cause of ferritin accumulation in the patient cells. Autophagy activity in patient cells was reduced to approximately 50% of that observed in the healthy cells. This indicates the *WDR45* gene expression is important for autophagy. However, impairment of iron metabolism has not been reported in response to the altered expression of major autophagy-related genes and we did not detect any significant changes in levels of iron and its related molecules in *ATG5*-deficient HeLa cells. This suggests that the involvement of an unknown mechanism. As ferritin accumulated and ferritin degradation was significantly reduced in the patient cells, we focused on iron metabolism attributed to ferritinophagy. In ferritinophagy, a cargo receptor, NCOA4, binds to the ferritin heavy chain and directs ferritin to the isolation membrane. NCOA4 deficiency leads to an inhibition of ferritinophagy and increases basal ferritin levels.^20,32^ Surprisingly, the protein expression of NCOA4 was significantly reduced in patient cells compared to that in healthy cells. In addition, the expression of NCOA4 protein was restored after the *WDR45* gene transfer. Other phenotypes such as the protein levels of ferritin, DMT1 and FPN were also restored to the levels observed in the healthy cells. These findings imply that ferritin accumulated due to the disruption of autophagy, especially that of ferritinophagy, in patients with SENDA/BPAN. *Ncoa4*-null mice displayed increased levels of ferric iron and ferritin, decreased levels of FPN in the liver, spleen, bone marrow and duodenum, and reduced ferritinophagy in mouse embryonic fibroblasts.^40^ These phenotypes are similar to the results observed in this study. These results indicate that dysregulation of iron metabolism is due to the reduced expression of NCOA4 in *WDR45* variants. However, forced expression of NCOA4 did not restore the ferritin levels in the patient cells. The qRT-PCR analysis showed no difference in NCOA4 mRNA levels between the patients and the healthy control. Moreover, NCOA4 colocalized with WDR45-EGFP in the control cells. These results suggest that WIPI4 may be involved in the stabilization or post-translational modification of NCOA4 proteins and binds with NCOA4 to degrade ferritin, although the precise mechanism remains unknown.

CNS-specific *Wdr45* knock-out mice exhibited extensive axon swelling with abundant swollen mitochondrial accumulation in these axons.^35^ In addition, patient-derived fibroblasts have previously demonstrated abnormal mitochondrial morphologies, decreased mitochondrial membrane potential and reduced production of ATP.^23^ These findings suggest that *WDR45* variants induce mitochondrial impairment; however, the mechanisms of this impairment remain to be elucidated.^41^ In this study, mitochondrial ATP synthesis was reduced in the patient cells. Therefore, we considered mitochondrial impairment may be attributed to the reduced ferrous iron content. Mitochondria are one of the main organelles that utilize ferrous iron for biochemical reactions such as the synthesis of heme and the production of ATP.^42^ Thus, insufficient levels of ferrous iron would interfere with mitochondrial function. Reportedly, HeLa cells with *NCOA4* knockdown were unable to maintain intracellular ferrous iron levels due to the inability of ferritin degradation, resulting in abnormalities in the mitochondrial respiratory chain activity.^43^ As shown in this study, intracellular ferrous iron content was decreased in patient cells, which may lead to the disruption of mitochondrial function. In addition, due to inhibited autophagic activity, impaired mitochondria that are normally degraded by autophagy could accumulate.

In the present study, we investigated the changes in *WDR45*/WIPI4 expression under autophagy induction. The mRNA expression of *WDR45* was mildly increased under starvation conditions with or without Baf A1 in both the control and patient cells. The protein expression of WIPI4 was decreased under starvation condition and recovered under starvation condition with Baf A1 in the control cells. This suggests that *WDR45* demand increased during autophagy induction and WIPI4 undergoes autophagic degradation. Furthermore, the autophagy activities were decreased to approximately 50% of that of the healthy control. The results are consistent with previous reports that *WDR45*/WIPI4 is a necessary molecule for autophagy.^2,4^

Quantitative proteomic analysis showed that ER proteins accumulated in *Wdr45* KO mice. This suggests that *Wdr45* deficiency resulted in increased ER stress and impaired ER quality control.^44^ In our study, protein expression of protein disulphide isomerase (PDI), one of the ER stress markers was increased in the patient cells (Supplementary Fig. 13). Since autophagy activity was decreased in the patient cells, unnecessary proteins and old organelles are not sufficiently degraded and removed. Therefore, it is considered that dysfunctional ER accumulate, and unfolded proteins cannot be removed, which leads to ER stress. Therefore, it is reasonable that ER stress also harms the patient cells.

Next, we explain why autophagic activity remains in patient cells. WIPI4 is one of the four WD40 repeat-containing proteins that interact with phosphoinositides (WIPI proteins family). WIPI proteins contain WIPI1, WIPI2, WIPI3/*WDR45B* and WIPI4/*WDR45*. *WDR45B* and *WDR45* are thought to have a redundant function. *Wdr45b* or *Wdr45* single KO mice exhibit impaired cognitive function and pathological changes in the central nervous system. However, double-deficient mice of *Wdr45b* and *Wdr45* die within one day of birth.^45^ In patients 1, 3 and 4, although WIPI4 was rarely expressed, autophagy activity was maintained at approximately 50% of that in control cells. The remaining 50% autophagic activity may depend on WIPI3 function.

At present, we have no curative treatment for SENDA/BPAN. Since patients with SENDA/BPAN show iron deposition in the basal ganglia, which is a characteristic feature,^1,3,4^ and increased ferric or total iron content in patient fibroblasts,^22,23^ it was believed that increased levels of iron may harm the cells via the production of ROS.^46^ Thus, iron chelators that reduce intracellular iron levels have been considered as candidates for SENDA/BPAN treatment. However, according to two clinical case reports, parkinsonism-like symptoms progressed and other symptoms such as anorexia, insomnia, restlessness and agitation appeared in patients treated with iron-chelator deferiprone. However, these symptoms improved after the discontinuation of deferiprone ^47,48^

This study revealed that the intracellular iron accumulation involved the ferric iron stored in ferritin, while the ferrous iron essentially required for intracellular biochemical reactions was insufficient. The progression of symptoms due to iron chelation therapy can be the result of decreased ferrous iron content. Therefore, a treatment strategy that increases intracellular ferrous iron content, i.e. increased ferritin degradation by improving ferritinophagy activity, is desirable. In this study, we measured autophagic activity using a fluorescent probe and found a notable difference between patients and healthy participants. From the viewpoint of autophagy, drug screening using cells transferred with a fluorescent probe in this study could be useful. We also confirmed that the *WDR45* gene transfer restored NCOA4 protein expression and iron metabolism. Since SENDA/BPAN is a single gene disease, gene therapy is also a promising candidate for future treatments. In addition, we need to evaluate more samples to examine the potential of gene therapy owing to the limited sample size used in this study.

**Figure 7:**
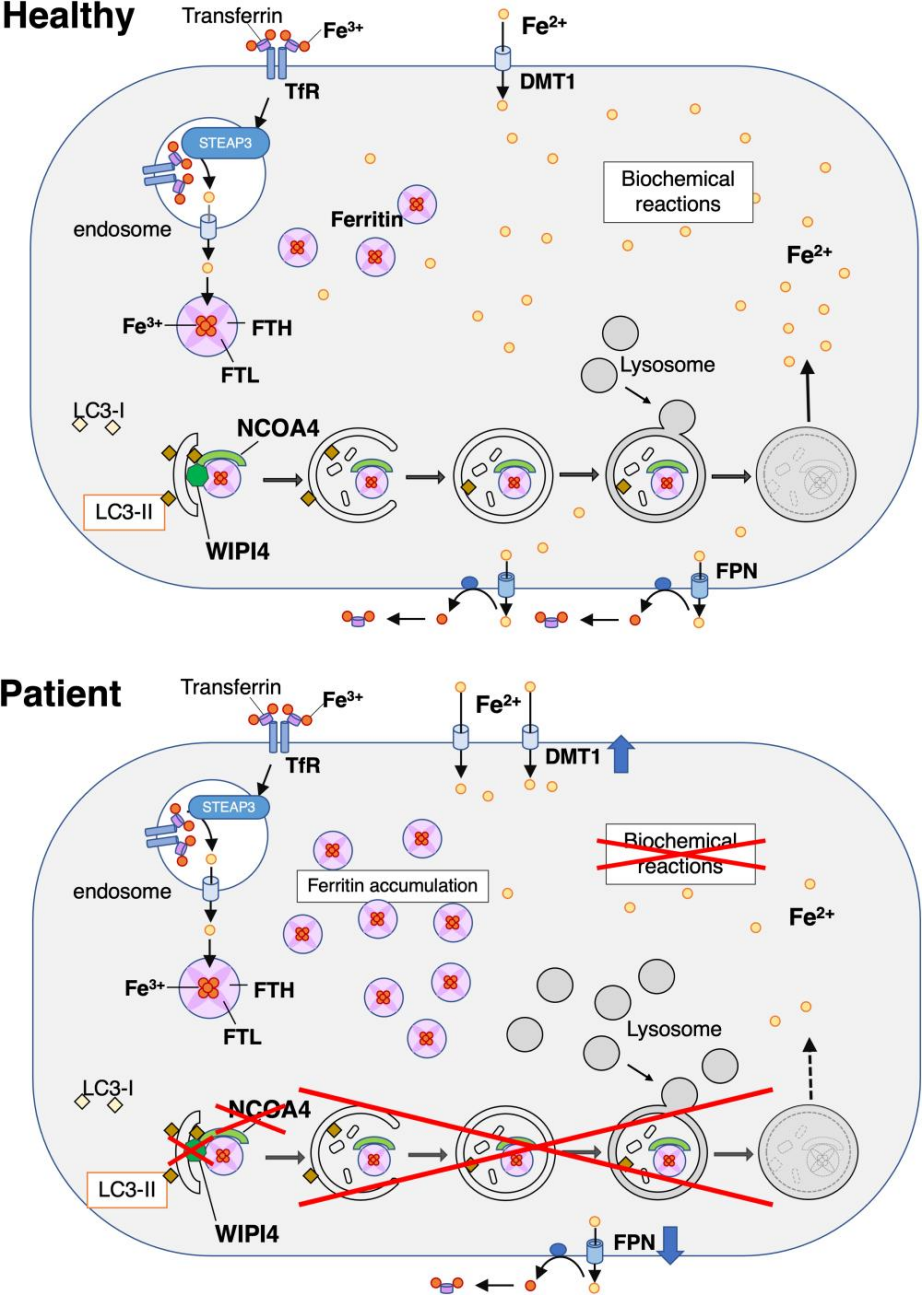
**Iron metabolism in healthy and SENDA/BPAN patient cells.** (**HeaIthy**) Iron metabolism in healthy cells. Ferrous iron enters cells from the extracellular space via DMT1, while ferric iron combined with transferrin enters via the transferrin receptor by endocytosis. Ferric iron is reduced to ferrous iron by STEAP3, and ferrous iron is then released into the cytoplasm. Intracellular ferrous iron is used for essential biochemical reactions. Excess ferrous iron is exported by FPN or oxidized in the ferritin heavy chain, changed to a non-toxic iron form, ferric iron and stored in the ferritin light chain. Depending on the demand of intracellular ferrous iron, ferritin is degraded via ferritinophagy and ferric iron included in ferritin is released as ferrous iron into the cytoplasm. (**Patient**) Impaired iron metabolism in patient cells. NCOA4 expression is decreased by deprivation of WIPI4. NCOA4-mediated ferritinophagy is impaired and leads to the accumulation of ferritin. The decreased supply of ferrous iron from ferritinophagy impacts the essential biochemical reactions. The expression of DMT1 increases and that of FPN decreases to improve the supply and reduce the export of ferrous iron.

In conclusion, we provide evidence that the *WDR45* variants impaired not only autophagy but also ferritinophagy by markedly reducing NCOA4 protein expression in patients with SENDA/BPAN. Consequently, ferric iron was accumulated, and the levels of the ferrous iron, which is necessary for biochemical reactions, was low. The insufficiency of ferrous iron led to cytotoxicity, which contrasts with previous findings. A graphical summary is shown in Fig. 7. The *WDR45* gene transfer by AAV vector could be a potential treatment strategy that can improve iron metabolism and the maintenance of cellular homeostasis, including mitochondrial function.

## Supplementary Material

fcac304_Supplementary_Data

## Data Availability

Data supporting the findings from this study are available within the article file and its supplementary information. Any other raw data or non-commercial material used in this study are available from the corresponding author upon reasonable request. Source data are provided with this paper. Reference sequences are available from the GenBank for Homo sapiens *WDR45* transcript variant 1 mRNA (NM_007075.3) and WIPI4 isoform 1 (NP_009006.2), and variant 2 mRNA (NM_001029896.2) and WIPI4 isoform 2(NP_001025067.1), which has an alternate 5′ UTR and lacks a 3-nt segment in the CDS, as compared with variant 1.

## Abbreviations

AAV =: adeno-associated virus
ATP =: adenosine triphosphate
Baf A1 =: bafilomycin A1
BPAN =: β-propeller protein-associated neurodegeneration
CMV =: cytomegalovirus
CTSD =: cathepsin D
DMEM =: Dulbecco's modified Eagle's medium
DMT1 =: divalent metal transporter 1
ER =: endoplasmic reticulum
FPN =: ferroportin
hGAPDH =: human glyceraldehyde-3-phosphate dehydrogenase
HUMARA =: human androgen receptor
ITRs =: inverted terminal repeats
KO =: knock-out
LAMP2 =: lysosome-associated membrane protein 2
NBIA =: neurodegeneration with brain iron accumulation
NCOA4 =: nuclear receptor coactivator 4
OCR =: oxygen consumption rate
PFA =: paraformaldehyde
ROS =: reactive oxygen species
SEM =: standard error of the mean
SENDA =: static encephalopathy of childhood with neurodegeneration in adulthood
TfR =: transferrin receptor
TPP1 =: tripeptidyl peptidase 1
WDR45 =: WD repeat domain 45
WIPI =: WD repeat domain phosphoinositide interacting protein
WT =: wild-type
