# Supplementary material for: WDR45 variants cause ferrous iron loss due to impaired ferritinophagy associated with nuclear receptor coactivator 4 and WD repeat domain phosphoinositide interacting protein 4 reduction
Source: Brain Commun. 2022 Nov 23;4(6):fcac304. doi: 10.1093/braincomms/fcac304 (PMC9897194; doi:10.1093/braincomms/fcac304)
Supplement: fcac304_Supplementary_Data [file fcac304_Supplementary_Data.zip › Supplementary_figures.pdf]

Supplementary Figure 1

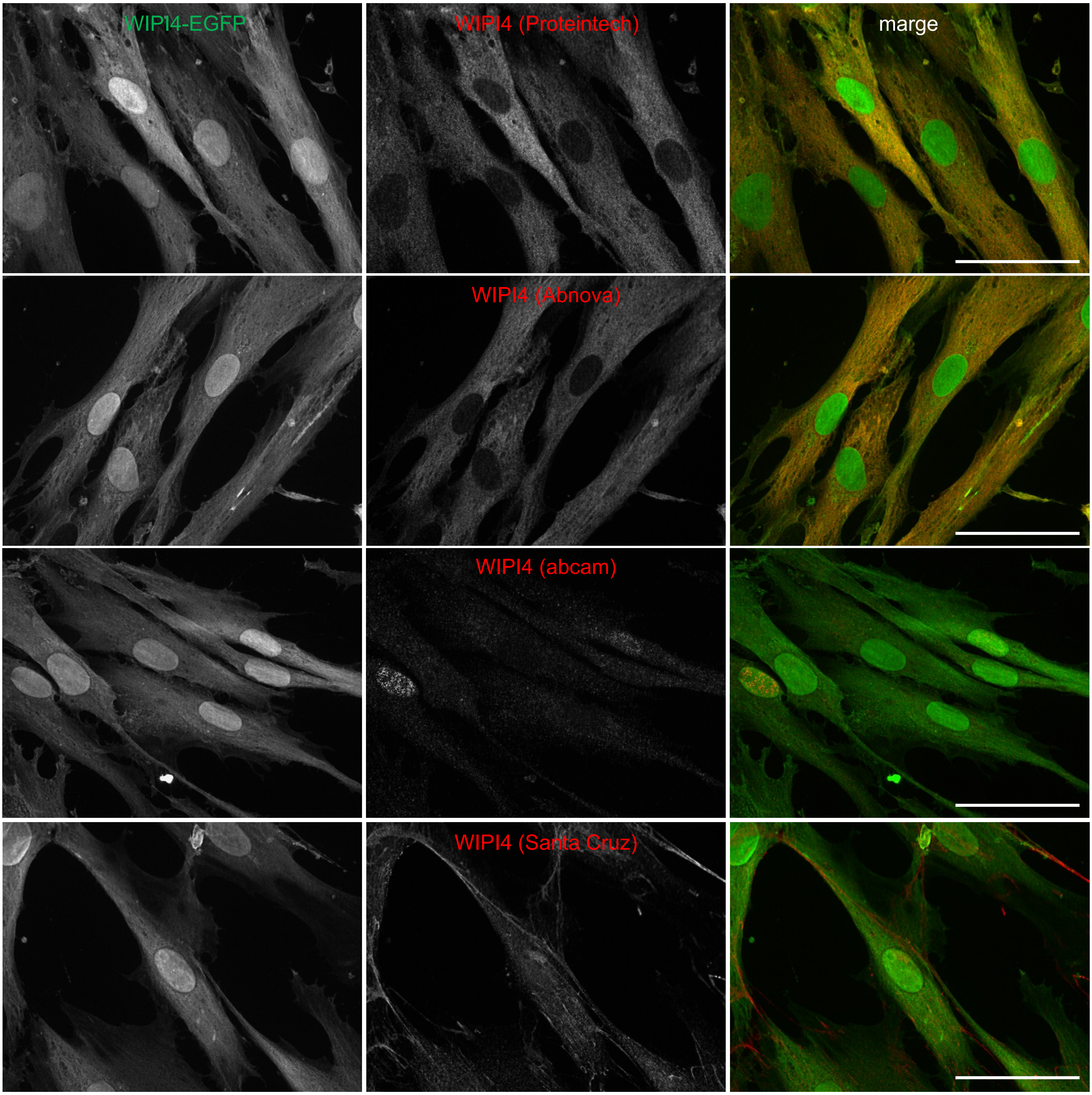

Supplementary Figure 1

Specificity of antibodies for *WDR45*/WIPI4 (immunofluorescence). The indicated stable fibroblast clones expressing WIPI4-EGFP were immunostained with all the available antibodies. Two antibodies could substantially detect WIPI4-EGFP expression. Scale bars = 50 μm

Supplementary Figure 2

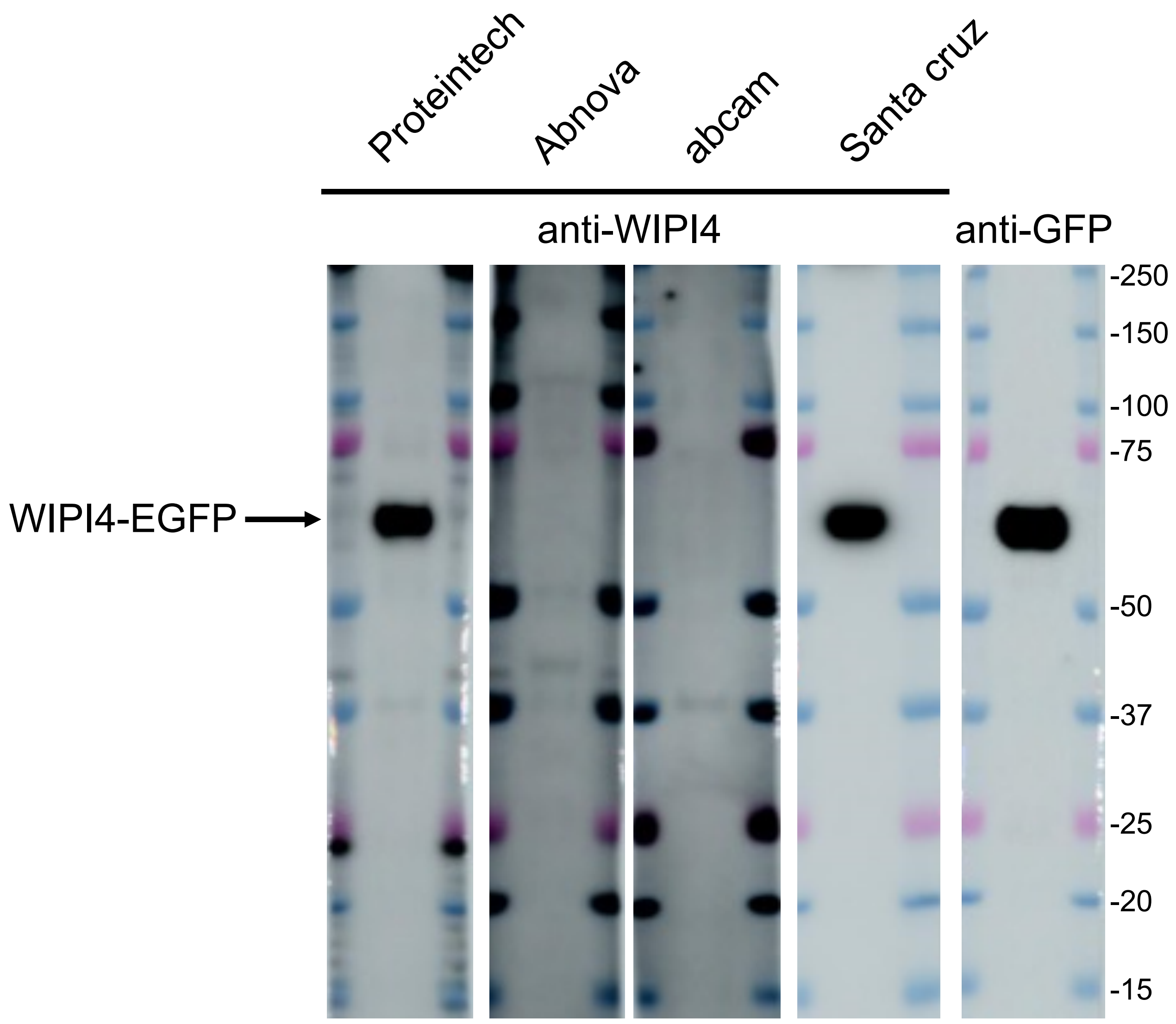

Supplementary Figure 2

Specificity of antibodies for *WDR45*/WIPI4 (immunoblotting). WIPI4-EGFP expression in stable fibroblasts expressing WIPI4-EGFP was confirmed via immunoblotting using all available antibodies. Two antibodies detected EGFP-tagged WIPI4; the intensity was comparable with that of the positive control, as assessed using an anti-GFP antibody. （Supplementary material for uncropped blots）

Supplementary Figure 3

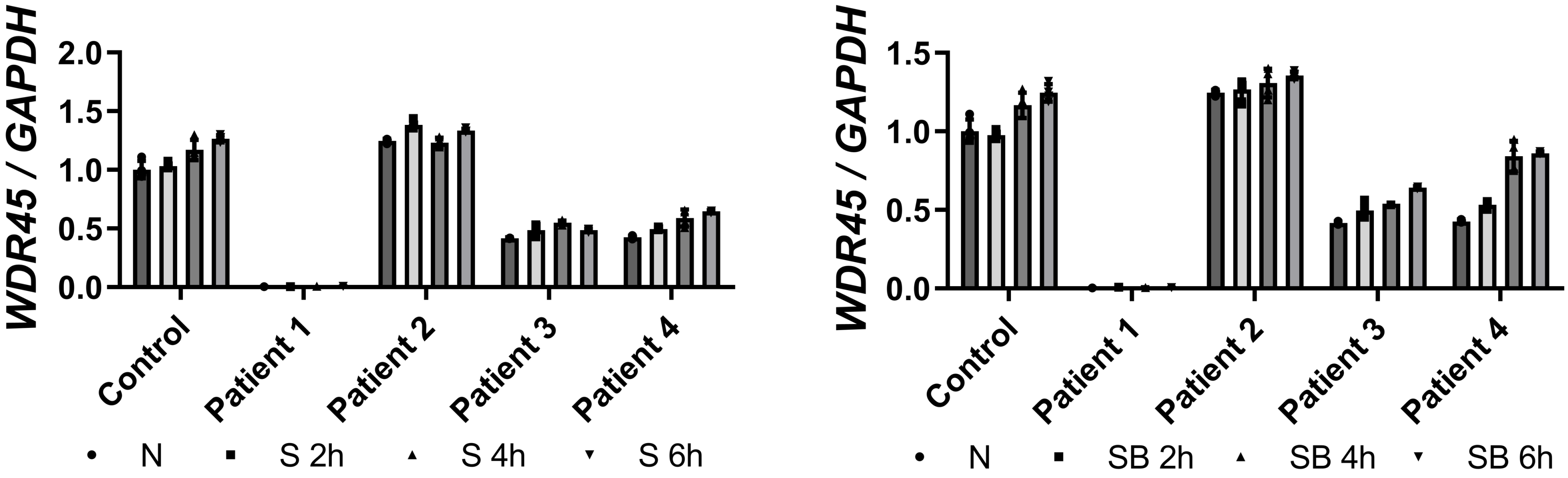

Supplementary Figure 3

The mRNA levels of *WDR45* over time under starvation conditions with or without Baf A1 treatment. *WDR45* mRNA expression ratio evaluated via qRT-PCR using TaqMan prove. The expression ratio was normalized to that of endogenous *hGAPDH* (**A**) during starvation without Baf A1 and (**B**) during starvation with BafA1. (n = 4) Data are represented as the mean ± SEM. N, nutrient condition; S, starvation condition; SB, starvation condition with Baf A1

Supplementary Figure 4

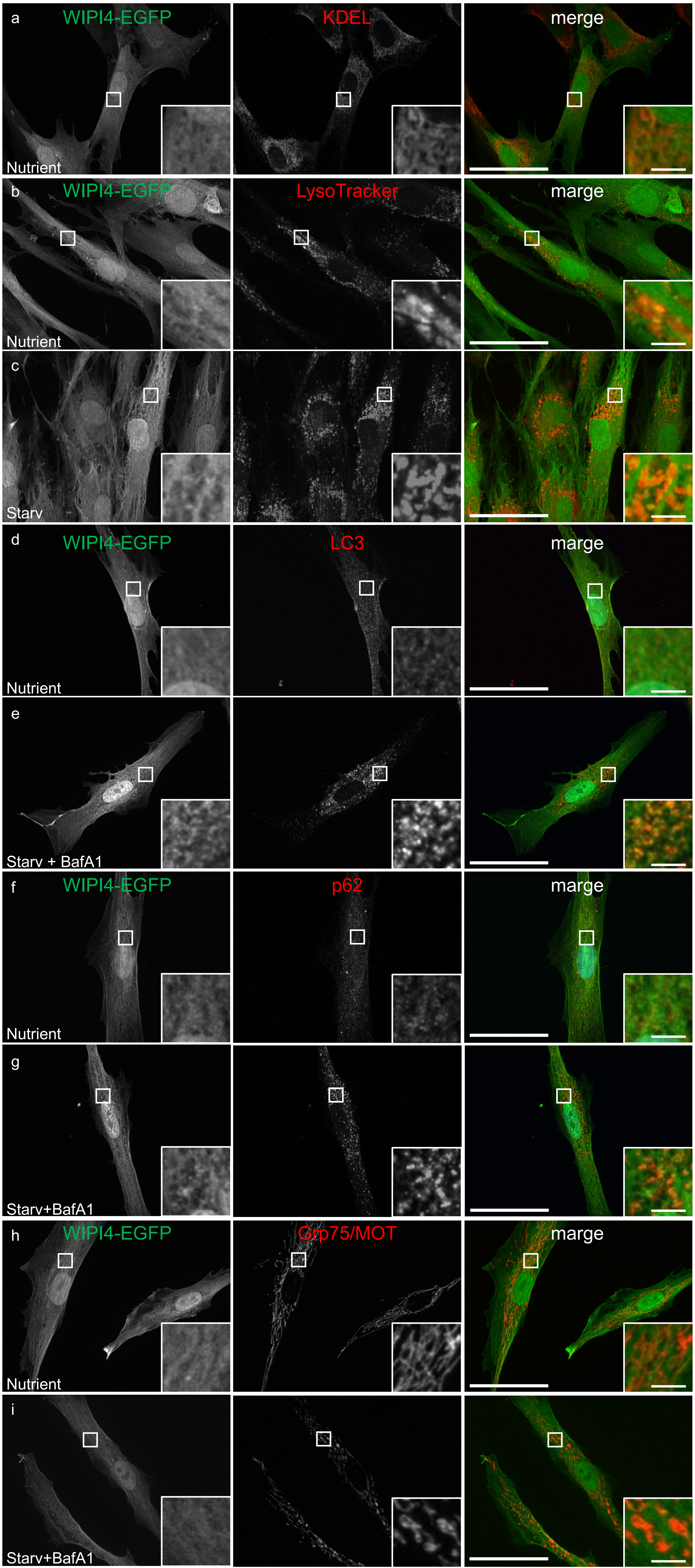

Supplementary Figure 4

Complete localization of WIPI4 within autophagosomes and partial localization with ER in fibroblasts. The expression of organelle markers and autophagy-related molecules in stable fibroblast clones expressing WIPI4-EGFP was investigated under conditions of nutrient availability, starvation, or lysosomal inhibition. **(A)** ER marker KDEL (red), **(B,C)** lysosomal marker Lysotracker (red), **(D,E)**, autophagosome marker LC3 (red), **(F,G)** autophagosome cargo protein p62 (red), **(H,I)** and mitochondrial marker Grp/MOT (red). ER marker KDEL motif partially colocalized with WIPI4 under conditions of nutrient availability. Lysosomes were in contact with WIPI4 under conditions of starvation. LC3 colocalized with WIPI4 under conditions of starvation with BafA1 treatment. p62 was in contact with WIPI4 under conditions of starvation with BafA1 treatment. Mitochondria did not colocalize with WIPI4. Scale bars = 50 μm and 5 μm in all insets. Nutrient, nutrient condition; Starv, Starvation condition; Starv + BafA1, lysosomal inhibition condition by starvation with BafA1 treatment.

Supplementary Figure 5

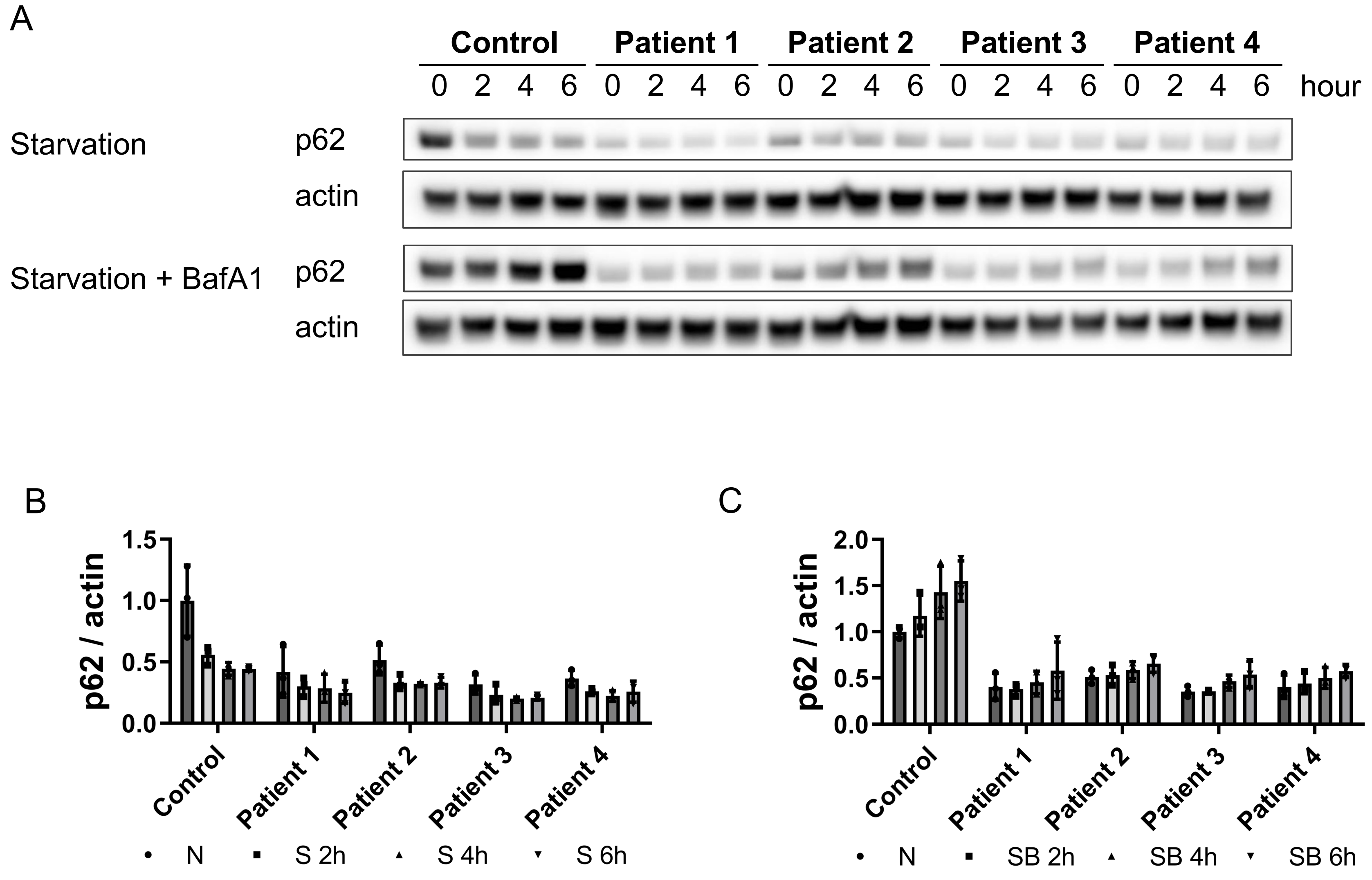

Supplementary Figure 5

Protein expression of p62 under starvation with or without Baf A1 treatment were evaluated for the indicated intervals using fibroblasts. (**A**) p62 was rapidly degraded in control fibroblasts; however, its degradation was suppressed in patient fibroblasts under starvation conditions. p62 accumulation was increased in control fibroblasts; however, it was suppressed in patient fibroblasts under starvation conditions with BafA1 treatment. Actin was used as the loading control. Data are representative of a minimum of three independent experiments. Actin was used as the loading control. (**B**,**C**) Quantitative expression ratio under starvation conditions with or without BafA1 treatment. (n = 3) Data are represented as the mean ± SEM. N, nutrient condition; S, starvation condition; SB, starvation condition with Baf A1. （Supplementary material for uncropped blots）

Supplementary Figure 6

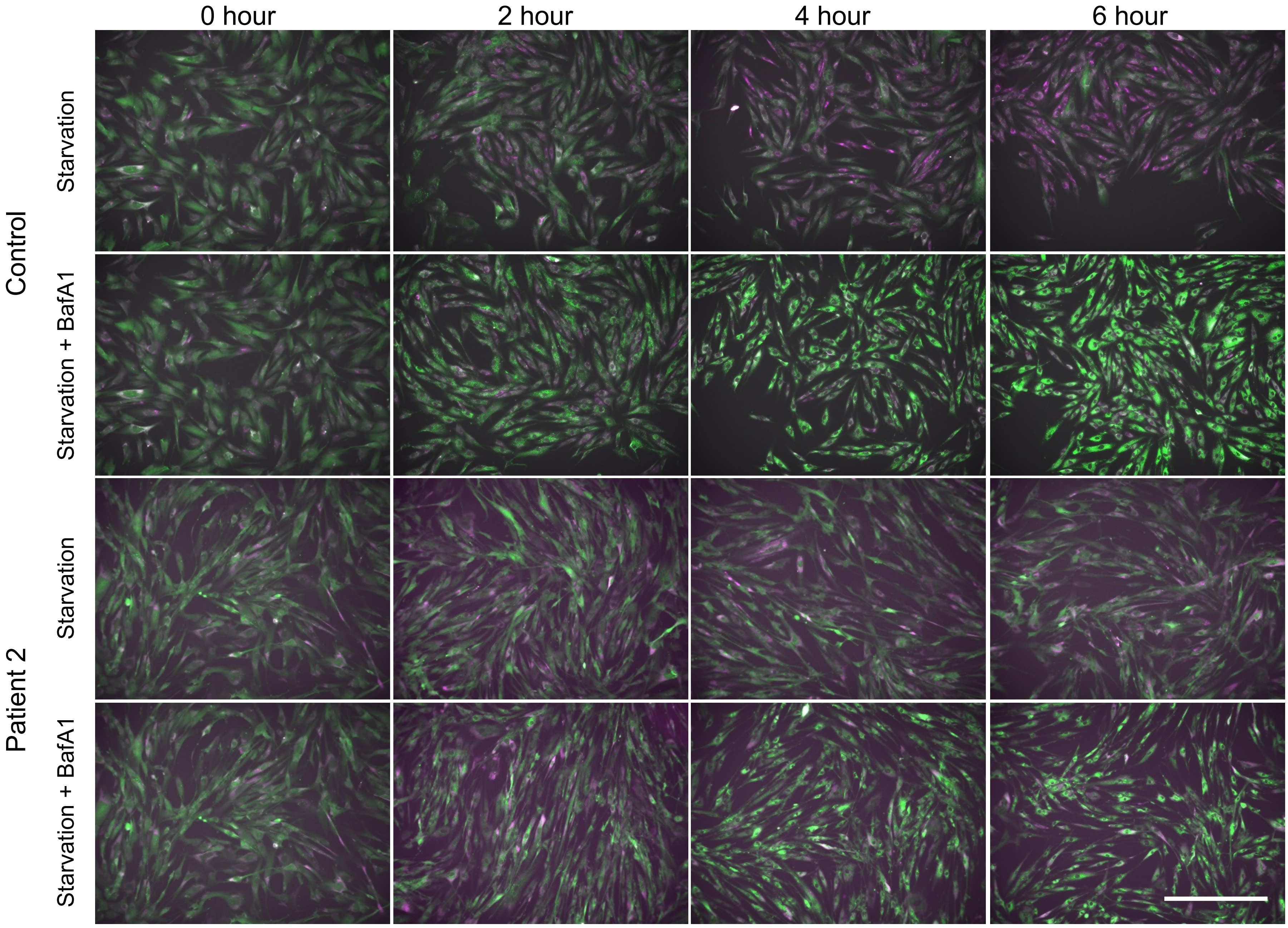

Supplementary Figure 6

In a stable fibroblast clone expressing the GFP-LC3-RFP probe, GFP-LC3 was degraded in a starvation-dependent manner but accumulated under conditions of starvation with BafA1 treatment in the control fibroblasts. However, GFP-LC3 was degraded to a lower degree in a starvation-dependent manner and also accumulated less under conditions involving starvation with BafA1 treatment in patient fibroblasts. The green signal represents high GFP/RFP and low autophagic activity, and the magenta signal represents low GFP/RFP and high autophagic activity. Scale bar, 200 μm.

Supplementary Figure 7

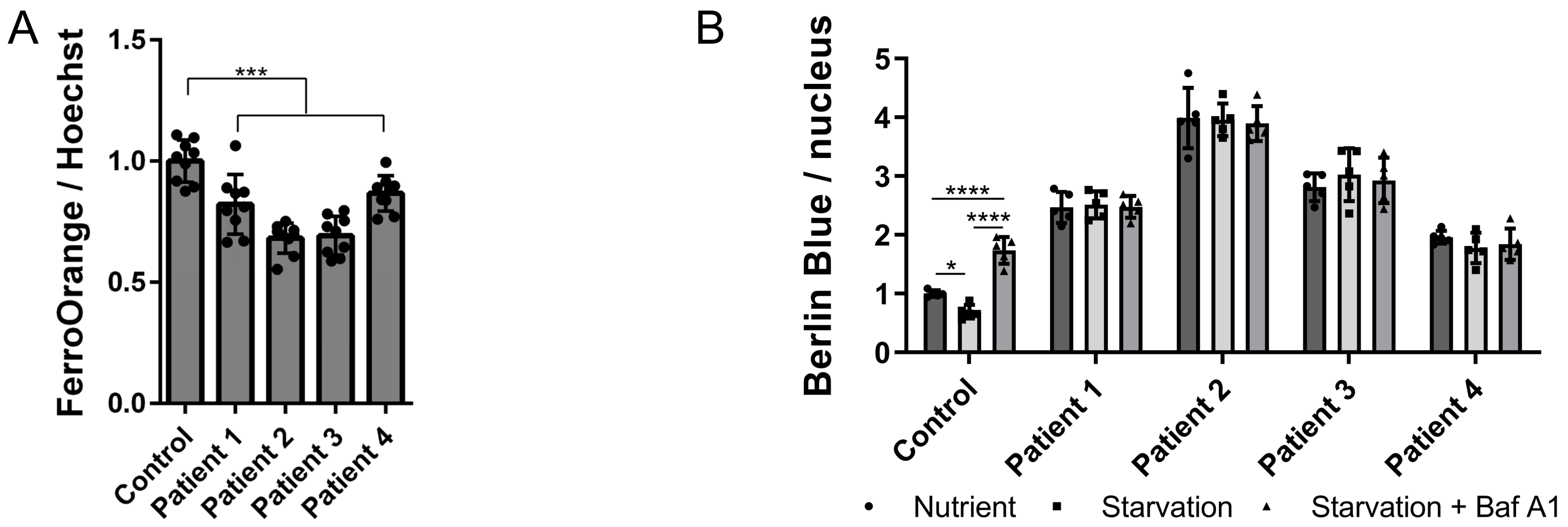

			Control	Patient 1	Patient 2	Patient 3	Patient 4
Nutrient	vs	starvation	0.019	0.9459	0.9917	0.6381	0.4205
Nutrient	vs	Starvation + Baf A1	<0.0001	0.9974	0.9193	0.8734	0.6882
starvation	vs	Starvation + Baf A1	<0.0001	0.9664	0.9609	0.9071	0.8885

Supplementary figure 7

Quantitative expression of Ferrous and ferric iron. **(A)** Fibroblasts were incubated with FerroOrange, which reacts with ferrous iron, and Hoechst. Fluorescent intensities were measured using a multimode plate reader. The intensity of FerroOrange was normalized by the intensity of Hoechst. The amount of ferrous iron decreased in the patient cells. One-way ANOVA with Wilcoxon test was used for analysis. (n=8, ***P <0.0001) **(B)** The intensities of Berlin blue staining were measured and normalized with the number of nucleus in each view. In control fibroblasts, ferric iron levels decreased under starvation conditions and increased under starvation conditions with Baf A1 treatment. In patient cells, there were no significant changes in the conditions. Tukey's HSD test was used for analysis. (n = 5, p values are listed in the table) Data are represented as the mean ± SEM.

Supplementary Figure 8

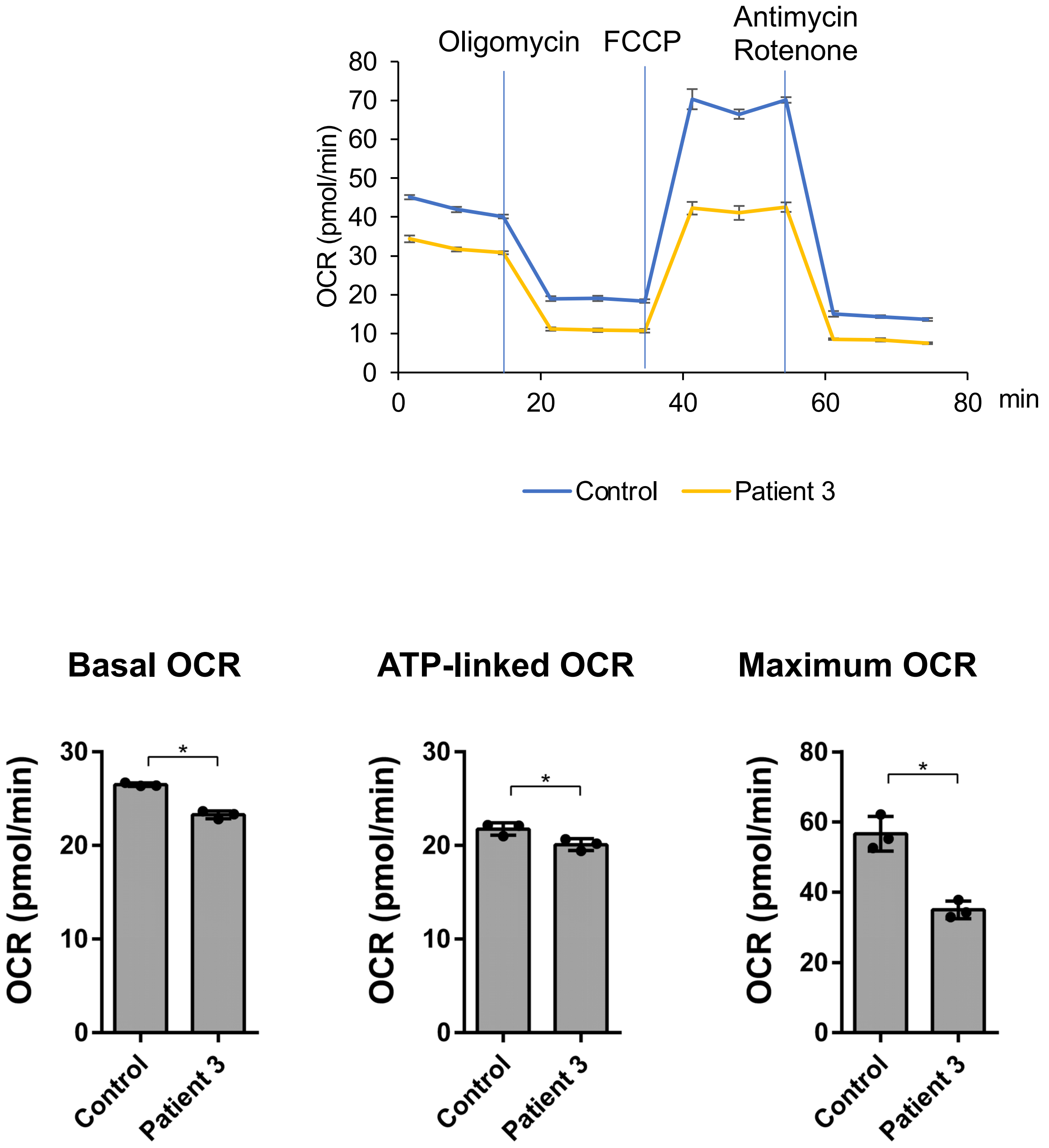

Supplementary Figure 8

Oxygen Consumption Rates (OCRs) of fibroblasts were measured using extracellular flux analyzer. In patient cells, basal OCR, ATP-linked OCR, Maximal OCR, and Spare Capacity were decreased compared with control cells. One-way ANOVA with Wilcoxon test was used for analysis. (n=3, Basal OCR: *P = 0.0495, ATP-linked OCR: *P = 0.0495, Maximum OCR: *P = 0.0495, Spare Capacity: *P = 0.0495) Data are represented as the mean ± SEM.

Supplementary Figure 9

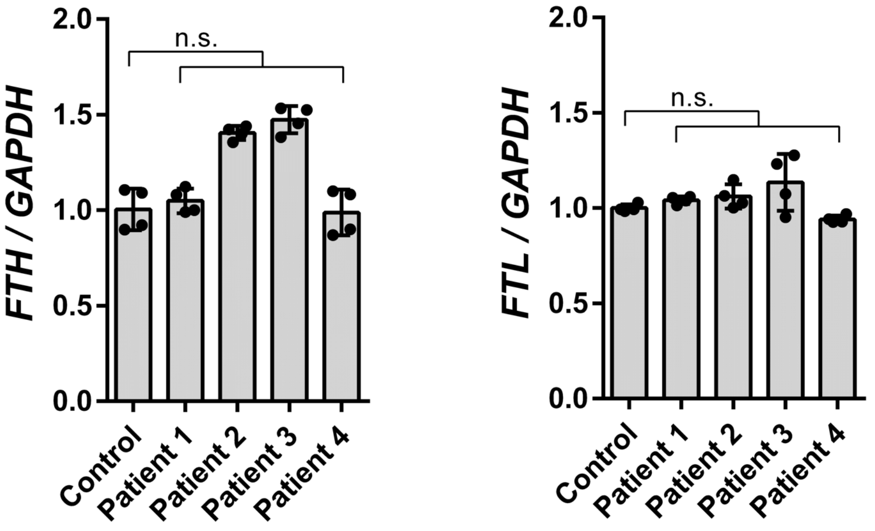

Supplementary figure 9

mRNA expressions of ferritin heavy and light chains were evaluated by qRT-PCR using TaqMan. The expression ratio was normalized to that of the endogenous human glyceraldehyde-3-phosphate dehydrogenase (*hGAPDH*). One-way ANOVA with Wilcoxon test was used for analysis. (n = 4, FTH: P = 0.1426, FTL: P = 0.3686) Data are represented as the mean ± SEM.

Supplementary Figure 10

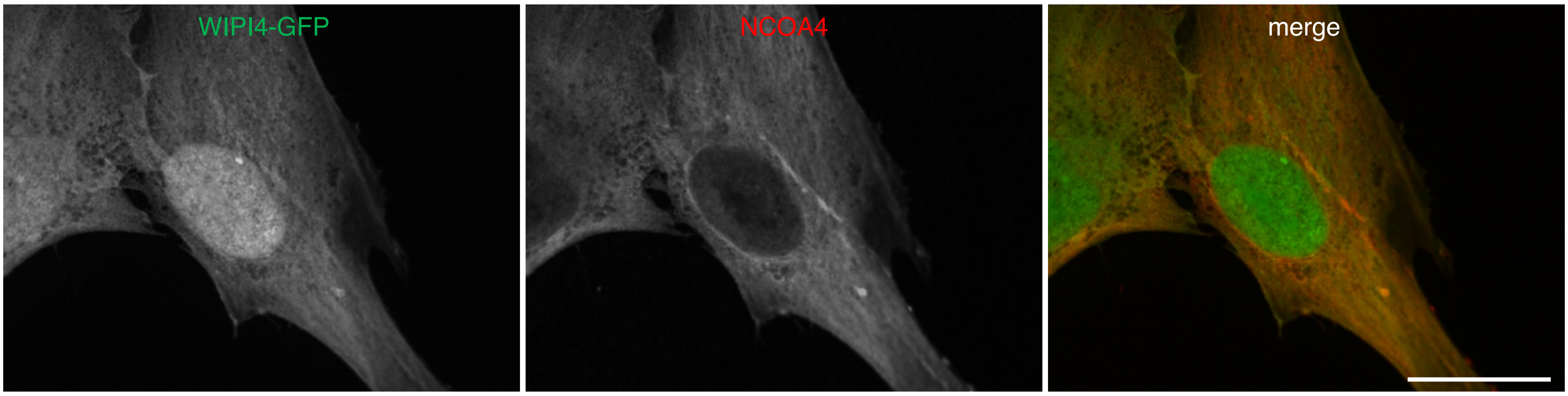

Supplementary Figure 10

WIPI4 colocalized with NCOA4 in fibroblasts. The expression of NCOA4 was investigated in stable fibroblast clones expressing WIPI4-EGFP under conditions of nutrient. WIPI4 completely colocalized with NCOA4 in the cytoplasm. Scale bars = 20 μm.

Supplementary Figure 11

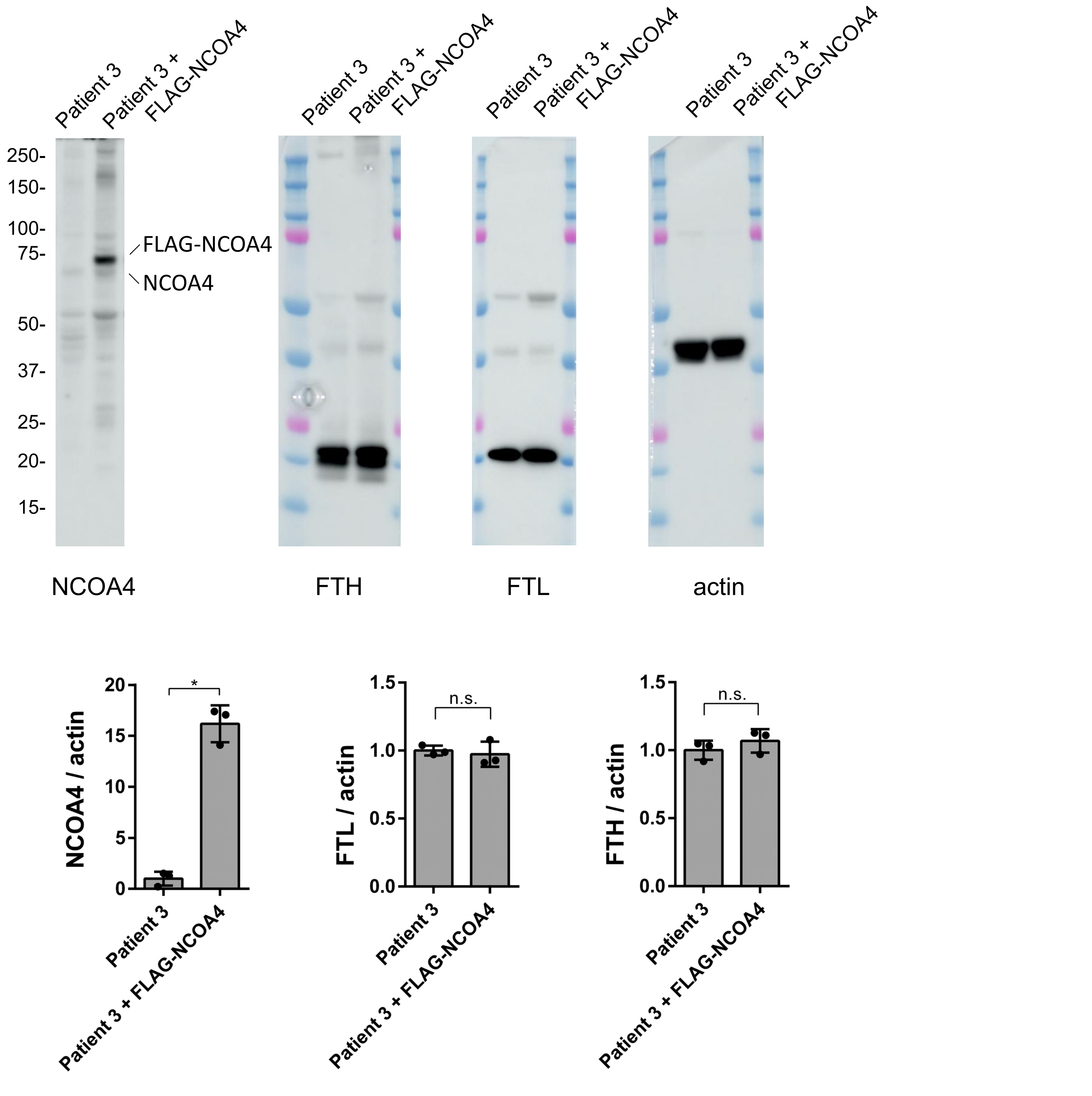

Supplementary Figure 11

Fibroblasts of patient 3 were transduced with FLAG-*NCOA4* using retrovirus. Protein expression of NCOA4, ferritin heavy and right chains were confirmed by immunoblotting. Actin was used as the loading control. Although NCOA4 protein was expressed in patient cells, ferritin heavy and right chain showed no significant changes by the gene transfer. One-way ANOVA with Wilcoxon test was used for analysis. (n = 3, NCOA4: P = 0.0495, FTH: P = 0.5127, FTL: P = 0.2752) Data are represented as the mean ± SEM.
（Supplementary material for uncropped blots）

Supplementary Figure 12

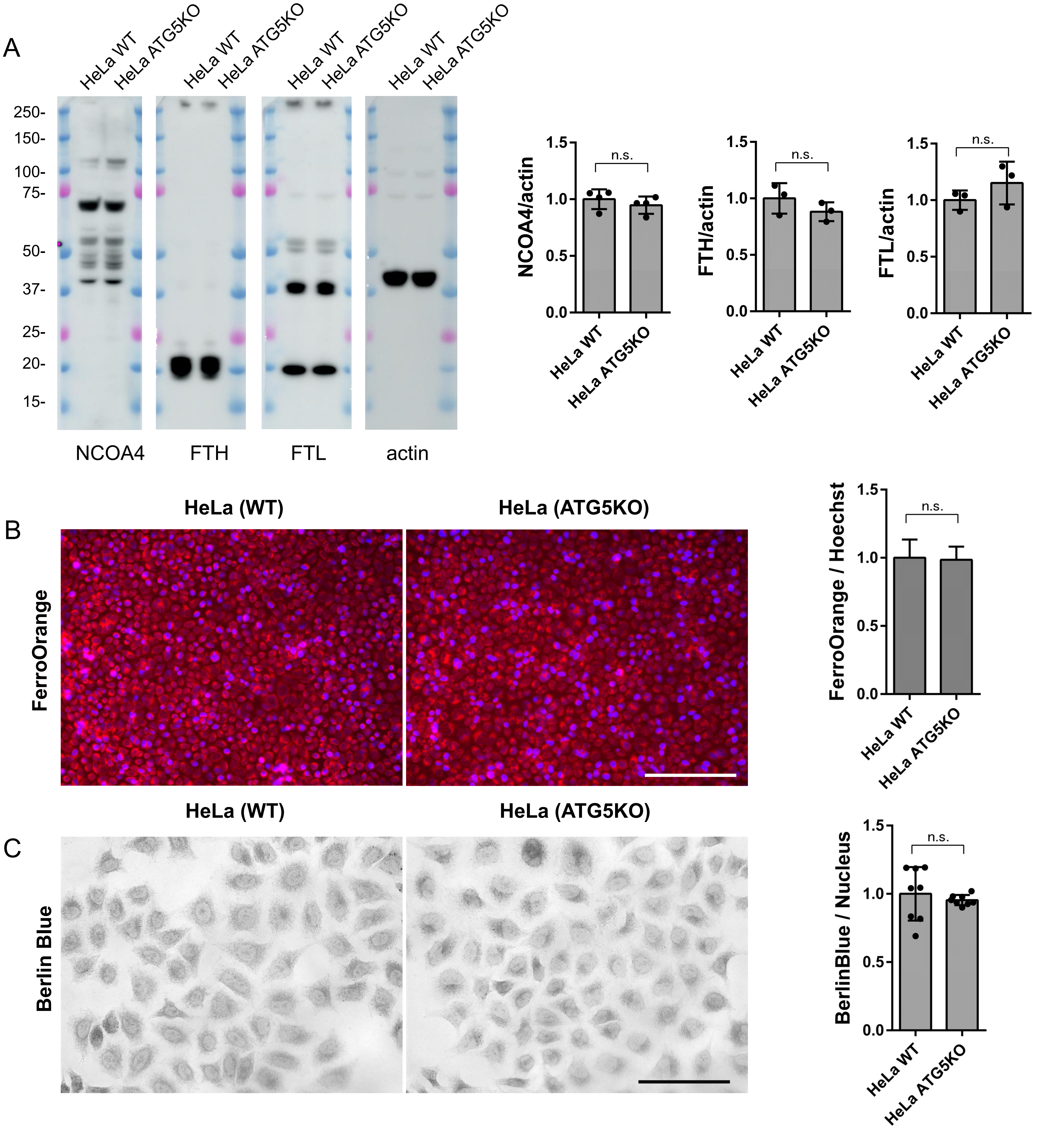

Supplementary Figure 12

The analysis of iron metabolism in the *ATG5*KO HeLa cells, the autophagy deficient cells **(A)** Protein expressions of NCOA4, ferritin heavy and right chains in HeLa cells were evaluated by immunoblotting. No significant differences were detected in the molecules between WT and *ATG5*KO cells. One-way ANOVA with Wilcoxon test was used for analysis. (n = 3, FTH: P = 0.2752, FTL: P = 0.2752, NCOA4: P = 0.2482) **(B)** HeLa cells were incubated with FerroOrange, which reacts with ferrous iron, and Hoechst. Fluorescent intensities were measured using a multimode plate reader. The intensity of FerroOrange was normalized based on the intensity of Hoechst. Scale bar = 200 μm There was no significant difference between WT and *ATG5*KO cells. One-way ANOVA with Wilcoxon test was used for analysis. (n = 11, P = 0.6931) **(C)** Berlin blue staining was performed using HeLa cells to confirm the amount of ferric iron. The intensity of Berlin blue stain was normalized based on the number of nucleus in each view. Scale bar = 100 μm There was no significant difference between WT and *ATG5* KO cells. One-way ANOVA with Wilcoxon test was used for analysis. (n = 8, P = 0.4008) Data are represented as the mean ± SEM.

Supplementary Figure 13

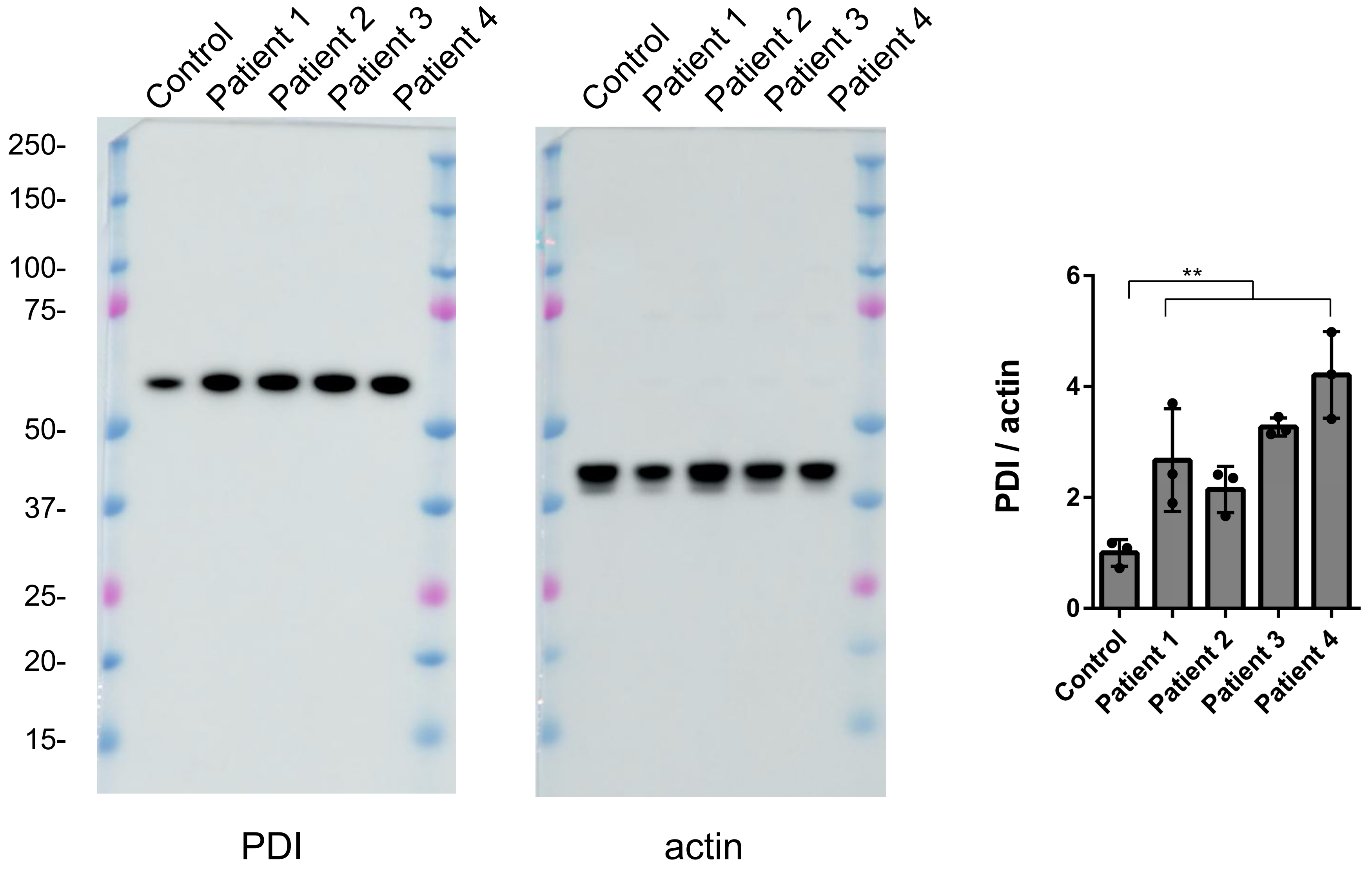

Supplementary Figure 13

The protein expression levels of PDI were analyzed by immunoblotting. In patient cells, PDI was increased compared to in the healthy control. Actin was used as the loading control. One-way ANOVA with Wilcoxon test was used for analysis. (n = 3, P = 0.0094) Data are represented as the mean ± SEM.
