# Supplementary material for: WDR45 variants cause ferrous iron loss due to impaired ferritinophagy associated with nuclear receptor coactivator 4 and WD repeat domain phosphoinositide interacting protein 4 reduction
Source: Brain Commun. 2022 Nov 23;4(6):fcac304. doi: 10.1093/braincomms/fcac304 (PMC9897194; doi:10.1093/braincomms/fcac304)
Supplement: fcac304_Supplementary_Data [file fcac304_Supplementary_Data.zip › Uncropped blots_Revised.pdf]

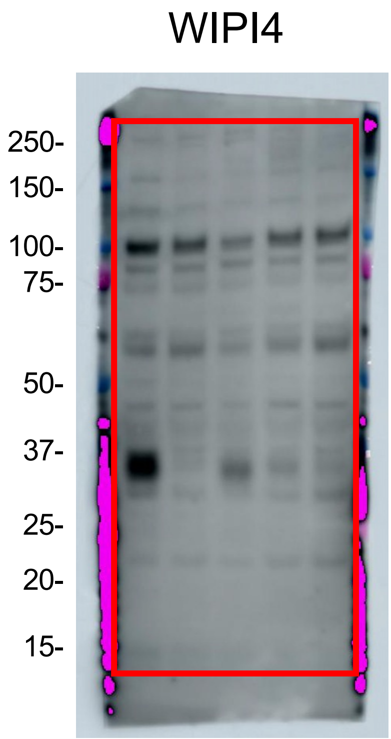

Uncropped blots for Figure 1C.

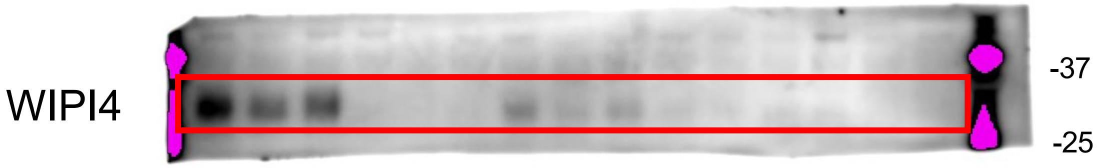

Uncropped blots for Figure 1D.

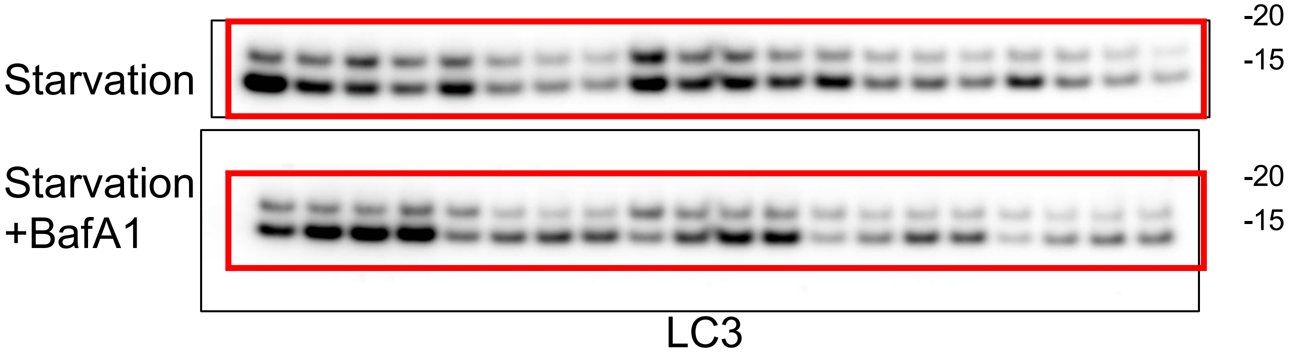

Uncropped blots for Figure 2A.

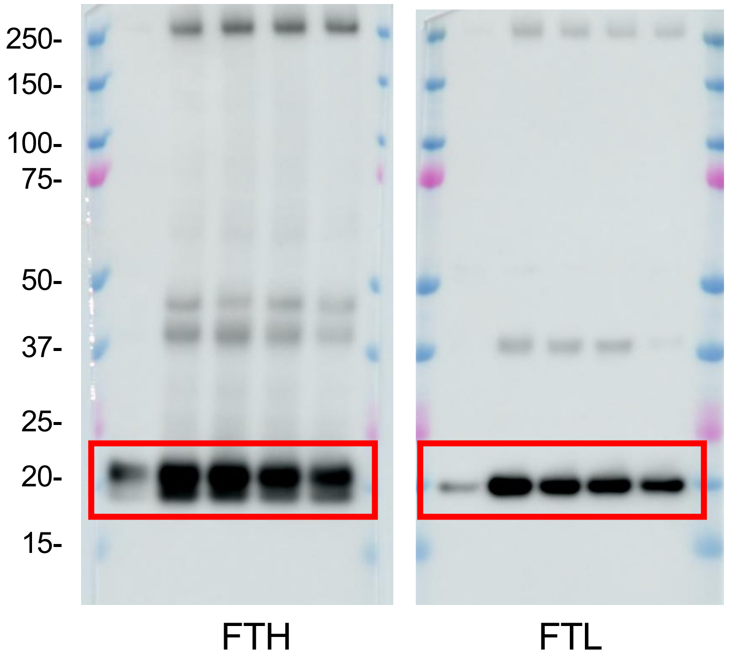

Uncropped blots for Figure 4A.

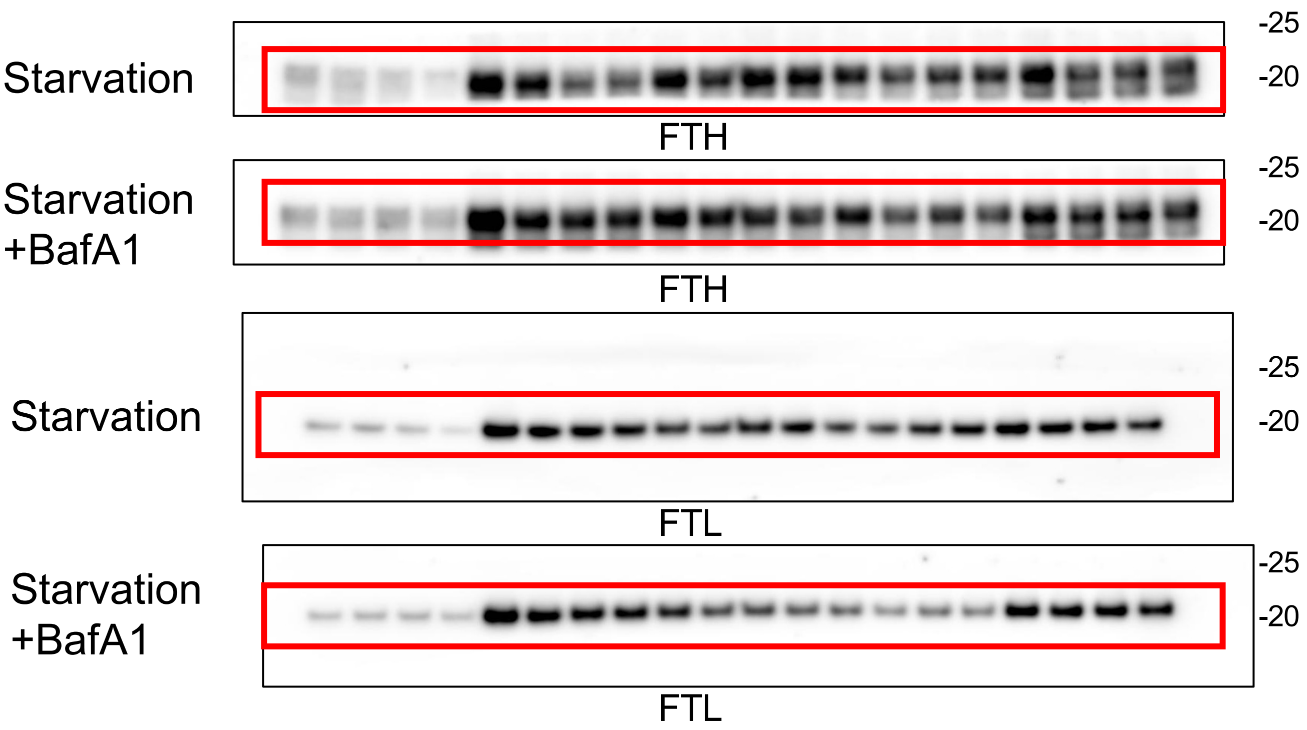

Uncropped blots for Figure 4C.

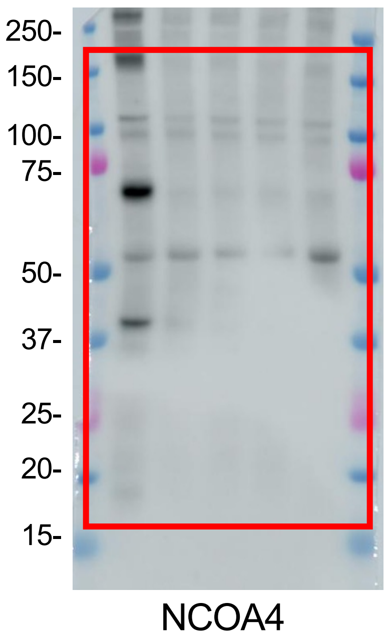

Uncropped blots for Figure 5A.

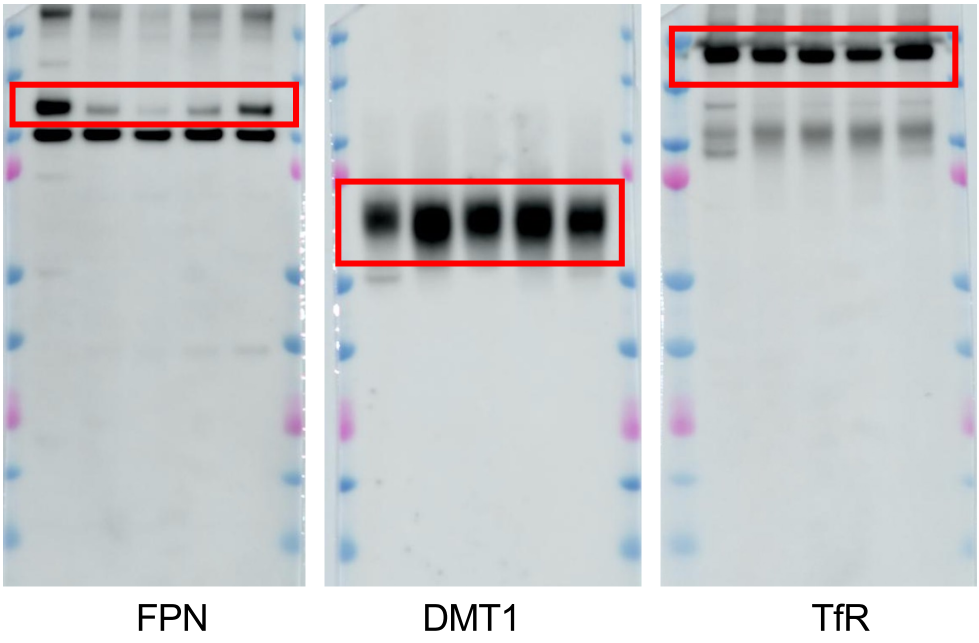

Uncropped blots for Figure 5C.

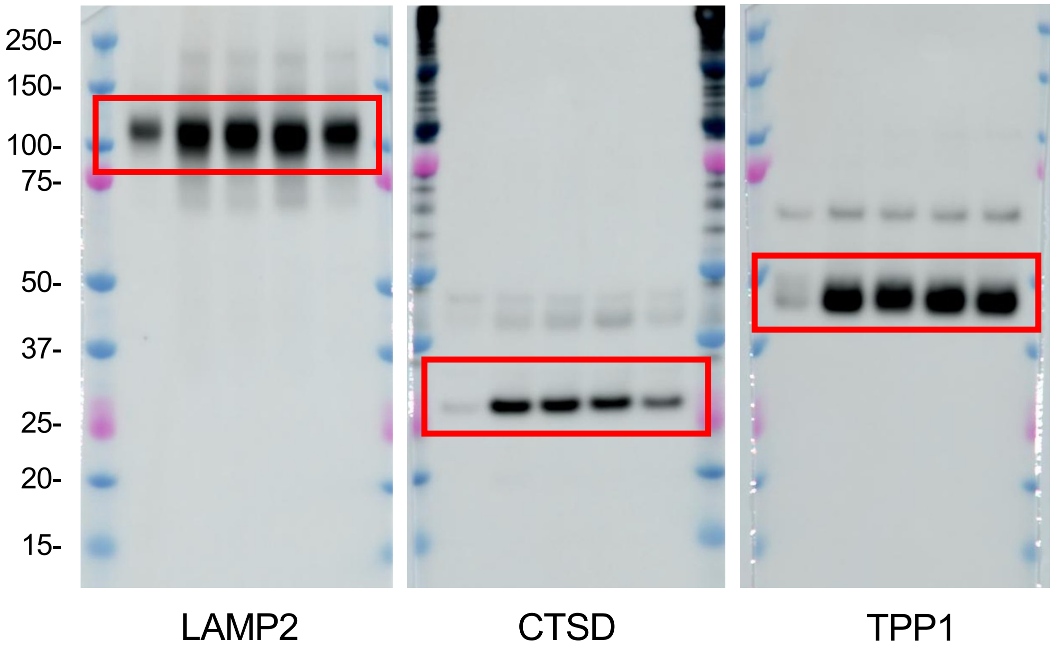

Uncropped blots for Figure 5D.

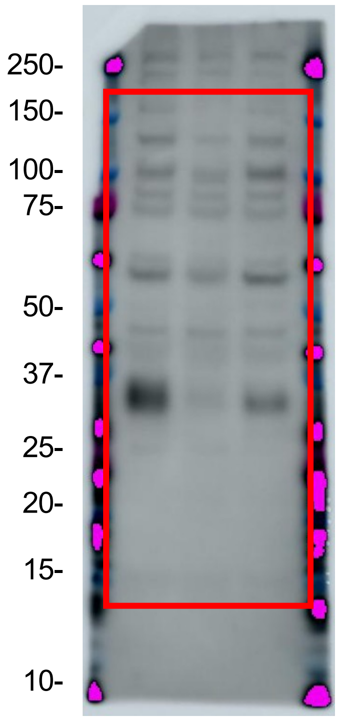

Uncropped blots for Figure 6C.

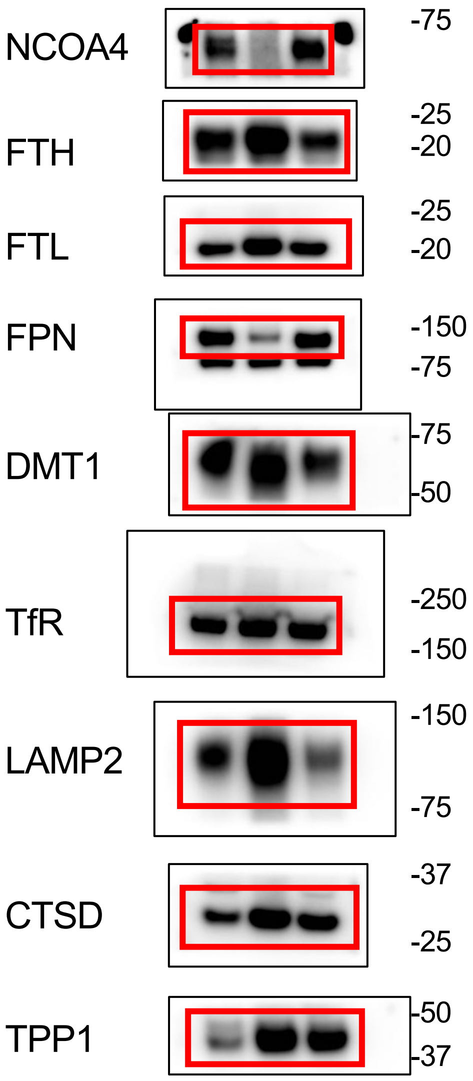

Uncropped blots for Figure 6D.

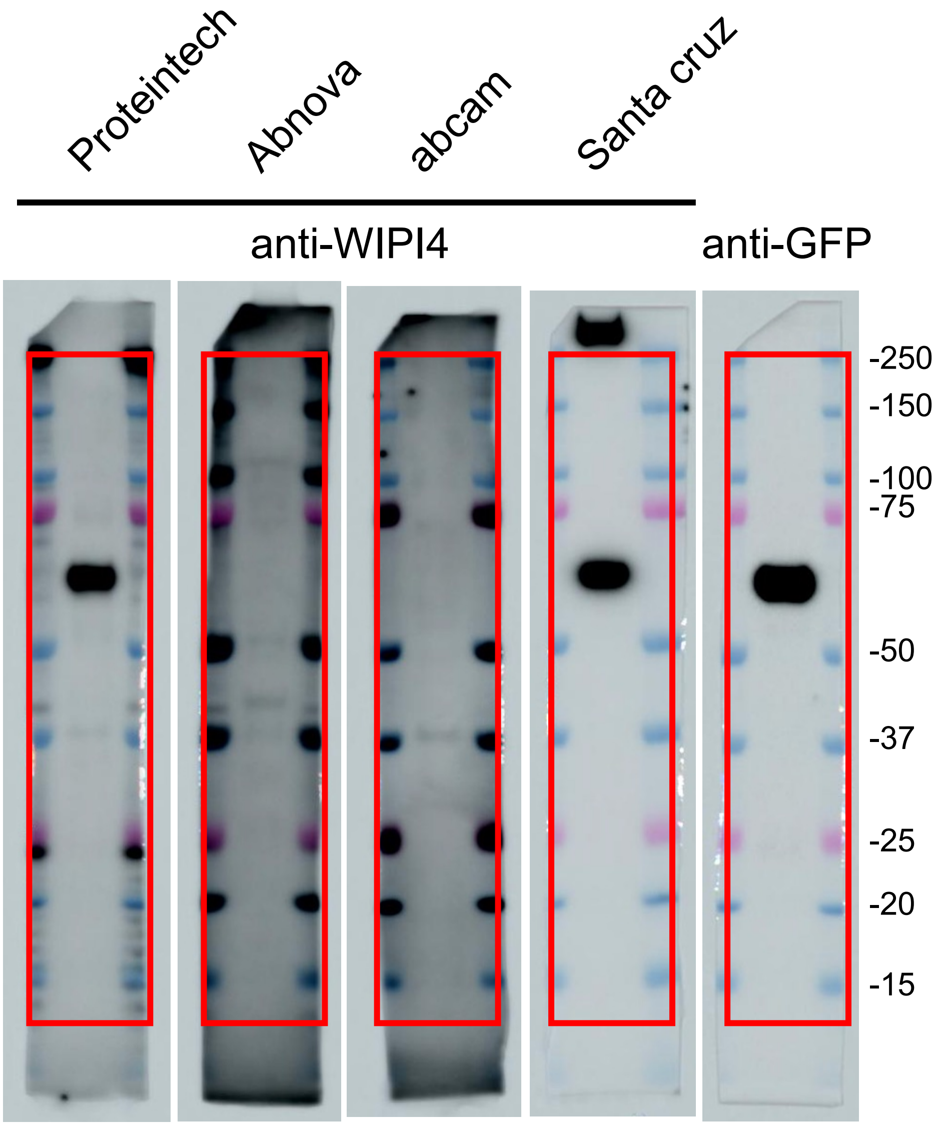

Uncropped blots for
Supplementary Figure 2.

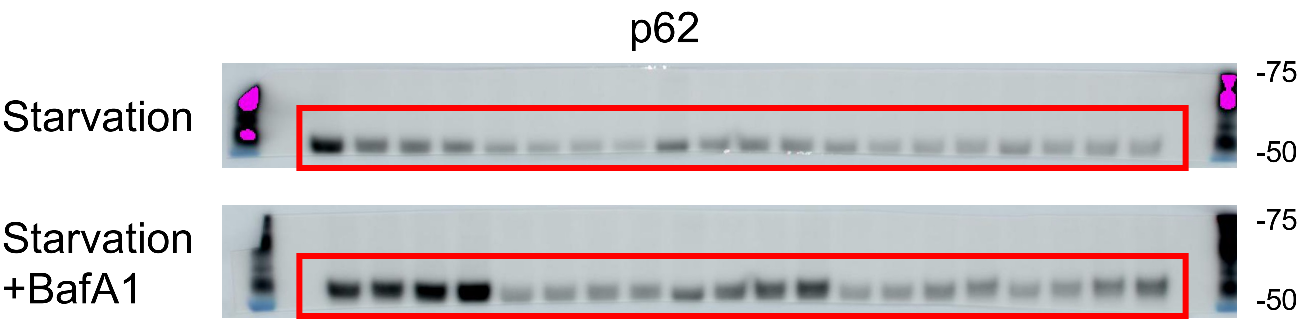

Uncropped blots for
Supplementary Figure 5.

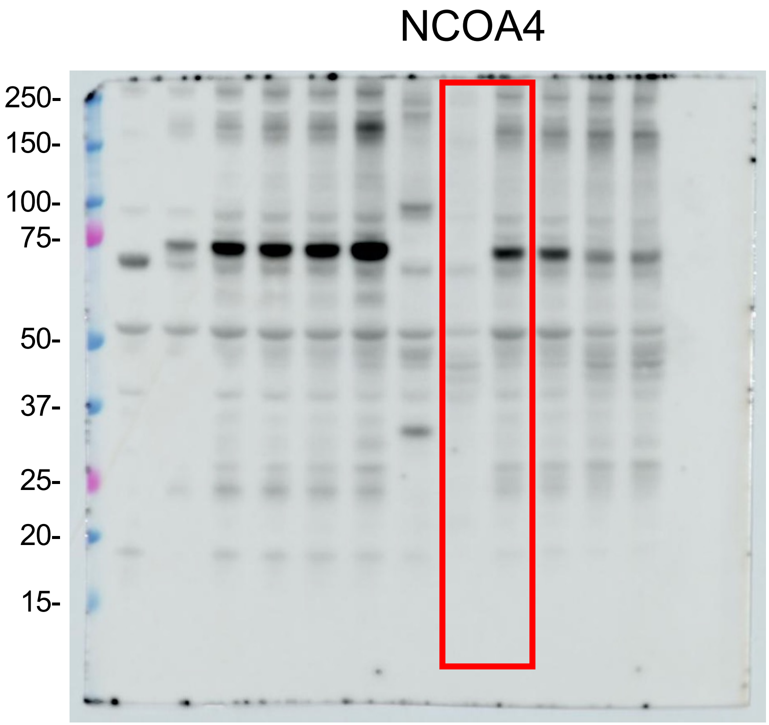

Uncropped blots for
Supplementary Figure 11.
