# Supplementary material for: WDR45 variants cause ferrous iron loss due to impaired ferritinophagy associated with nuclear receptor coactivator 4 and WD repeat domain phosphoinositide interacting protein 4 reduction
Source: Brain Commun. 2022 Nov 23;4(6):fcac304. doi: 10.1093/braincomms/fcac304 (PMC9897194; doi:10.1093/braincomms/fcac304)
Supplement: fcac304_Supplementary_Data [file fcac304_Supplementary_Data.zip › Supplementary_tables.doc]

**Supplementary information**

Supplementary Table 1

PCR conditions and primer sequences for mutation screening

| Exon | Amplicon size (bp) | Forward primer (5′→3′) | Reverse Primer (5′→3′) |
| --- | --- | --- | --- |
| 3-5 | 599 | GAGGAGGAAATCAGGTGAGGCAGAA | GAGGCCAGGAATCCGAGAAATCTG |
| 6-7 | 498 | AAGTAGGTTGAGGTCCCTGGGAAGC | TTCTTTCCTCATCTCTGCCCCTCTG |
| 8-12 | 1274 | TCCTCATCCAGCTCTGTCCATTCTG | GCTCAGCTCTGCAGTTCTGCCACT |
| Ex 8-12 internal sequencing primer | | | |
|  | | TCTGCGCTTCCAGTGATAAG | GTGGGGAGGGGGTACTCA |

The PCR comprised 40 cycles at 98 °C for 10 s, 64 °C for 20 s, and 68 °C for 1 min.

Supplementary Table 2

PCR conditions and primer sequences for X-inactivation analysis

| Analysis | Amplicon size（bp） | Forward primer (5′→3′) FAM-labeled | Reverse primer (5′→3′) |
| --- | --- | --- | --- |
| HUMARA | 291 | CCAGAATCTGTTCCAGAGCGTGC | CTCTACGATGGGCTTGGGGAGAAC |

The PCR comprised 35 cycles at 94 °C for 20 s, 65 °C for 20 s, and 72 °C for 20 s.

Supplementary Table 3

The primary and secondary antibodies for immunoblotting

| Antibody | Source | Catalog number | Dilution |
| --- | --- | --- | --- |
| Rabbit anti-Cathepsin D antibody | Thermo Fisher | PA5-72182 | 1:2,000 |
| Mouse anti-DMT1 antibody | Abnova | H00004891-M01 | 1:1,000 |
| Mouse anti-Ferritin Heavy chain antibody | Santa Cruz | sc-386594 | 1:5,000 |
| Mouse anti-Ferritin Light chain antibody | Santa Cruz | sc-74513 | 1:5,000 |
| Rabbit anti-Ferroportin antibody | ALPHA DIAGNOSIS | MTP11-A | 1:5,000 |
| Mouse anti-GFP antibody | Santa Cruz | sc-9996 | 1:200 |
| Mouse anti-LAMP2 antibody | NOVUS | NBP2-22217 | 1:5,000 |
| Rabbit anti-NCOA4 antibody | Thermo Fisher | A302-0272A | 1:500 |
| Mouse anti-NCOA4 antibody | Santa Cruz | sc-373739 | 1:100 |
| Mouse anti-Transferrin receptor antibody | Thermo Fisher | 13-6800 | 1:5,000 |
| Mouse anti-TPP-1 antibody | abcam | Ab5685 | 1:2,000 |
| Rabbit anti-WDR45 antibody | Proteintech | 19194-1-AP | 1:500 |
| Mouse anti-WDR45 antibody | Abnova | H00011152-B01P | 1:200 |
| Rabbit anti-WDR45 antibody | abcam | Ab240905 | 1:1,000 |
| Rabbit anti-WDR45 antibody | Santa Cruz | sc-398272 | 1:100 |
| Rabbit anti-p62 antibody | MBL | PM045 | 1:5,000 |
| Rabbit anti-PDI antibody | Cell signaling technology | #3501 | 1;10,000 |
| HRP-conjugated anti-β-actin antibody | MBL | PM053-7 | 1:5,000 |
| HRP-conjugated anti-LC3 antibody | MBL | M186-7Y | 1:5,000 |
| HRP-conjugated anti-mouse IgG antibody | Thermo Fisher | G-21234 | 1:5,000 |
| HRP-conjugated anti-rabbit IgG antibody | Thermo Fisher | G-21040 | 1:5,000 |

Supplementary Table 4

The TaqMan probes

| TaqMan | Assay ID |
| --- | --- |
| NCOA4 | Hs00428331_g1 |
| WDR45 | Hs01079049_g1 |
| FTH | Hs01694011_s1 |
| FTL | Hs00830226_gH |
| hGAPDH | Hs03929097_g1 |

Supplementary Table 5

The primary and secondary antibodies for immunofluorescence

| Antibody | Source | Catalog number | Dilution |
| --- | --- | --- | --- |
| Rabbit anti-LC3 antibody | MBL | PM036 | 1:1,000 |
| Rabbit anti-p62 antibody | MBL | PM045 | 1:500 |
| Mouse anti-Grp75/MOT antibody | abcam | ab2799 | 1:500 |
| Mouse anti-KDEL antibody | MBL | M181-3 | 1:1,000 |
| Rabbit anti-WDR45 antibody | Proteintech | 19194-1-AP | 1:500 |
| Mouse anti-WDR45 antibody | Abnova | H00011152-B01P | 1:200 |
| Rabbit anti-WDR45 antibody | abcam | Ab240905 | 1:1,000 |
| Rabbit anti-WDR45 antibody | Santa Cruz | sc-398272 | 1:100 |
| Rabbit anti-ferritin antibody | ROCKLAND | 200-401-090-0100 | 1:200 |
| Rabbit anti-NCOA4 antibody | Thermo Fisher | A302-272A | 1:100 |
| Alexa Fluor 488 goat anti-mouse IgG(H+L) | Thermo Fisher | A11029 | 1:500 |
| Alexa Fluor 594 goat anti-rabbit IgG(H+L) | Thermo Fisher | A11037 | 1:500 |

Supplementary Table 6

P-values of Figure 5

| Wilcoxon test P-value | |
| --- | --- |
| NCOA4 | 0.0094 |
| FPN | 0.0094 |
| DMT1 | 0.0094 |
| TfR | 0.8501 |
| LAMP2 | 0.0094 |
| CTSD | 0.0025 |
| TPP1 | 0.0094 |

Supplementary Table 7

P-values of Figure 6

| Tukey-HSD test P-values | | | |
| --- | --- | --- | --- |
|  | Control vs Patient 3 | Control vs Patient 3 + WDR45 | Patient 3 vs  Patient 3 + WDR45 |
| WIPI4 | < 0.0001 | 0.001 | 0.0012 |
| NCOA4 | 0.0007 | 0.0202 | < 0.0001 |
| FTH | 0.0004 | 0.1316 | < 0.0001 |
| FTL | 0.0308 | 0.9077 | 0.0189 |
| FPN | 0.0005 | 0.9229 | 0.0004 |
| DMT1 | 0.0338 | 0.4555 | 0.0082 |
| TfR | 0.9969 | 0.9059 | 0.9349 |
| LAMP2 | 0.0082 | 0.9992 | 0.0079 |
| CTSD | 0.0014 | 0.0308 | 0.048 |
| TPP1 | 0.0175 | 0.2431 | 0.1602 |
